# Three's a crowd – stabilisation, structure, and applications of DNA triplexes

**DOI:** 10.1039/d2sc01793h

**Published:** 2022-08-24

**Authors:** Maria Dalla Pozza, Ahmad Abdullrahman, Christine J. Cardin, Gilles Gasser, James P. Hall

**Affiliations:** Chimie ParisTech, PSL University, CNRS, Institute of Chemistry for Life and Health Sciences, Laboratory for Inorganic Chemical Biology F-75005 Paris France gilles.gasser@chimieparistech.psl.eu www.gassergroup.com; Department of Pharmacy, Chemistry and Pharmacy Building, University of Reading Whiteknights Campus Reading Berkshire RG6 6AD UK james.hall@reading.ac.uk; Department of Chemistry, University of Reading Whiteknights Reading RG6 6AD UK c.j.cardin@reading.ac.uk

## Abstract

DNA is a strikingly flexible molecule and can form a variety of secondary structures, including the triple helix, which is the subject of this review. The DNA triplex may be formed naturally, during homologous recombination, or can be formed by the introduction of a synthetic triplex forming oligonucleotide (TFO) to a DNA duplex. As the TFO will bind to the duplex with sequence specificity, there is significant interest in developing TFOs with potential therapeutic applications, including using TFOs as a delivery mechanism for compounds able to modify or damage DNA. However, to combine triplexes with functionalised compounds, a full understanding of triplex structure and chemical modification strategies, which may increase triplex stability or *in vivo* degradation, is essential – these areas will be discussed in this review. Ruthenium polypyridyl complexes, which are able to photooxidise DNA and act as luminescent DNA probes, may serve as a suitable photophysical payload for a TFO system and the developments in this area in the context of DNA triplexes will also be reviewed.

## Abstract 2

Triplex-forming oligonucleotides can target specific DNA sequences by binding in the duplex major groove. Chemical modifications and ligand binding have been explored, for use in a variety of biological applications.

## Graphical abstract

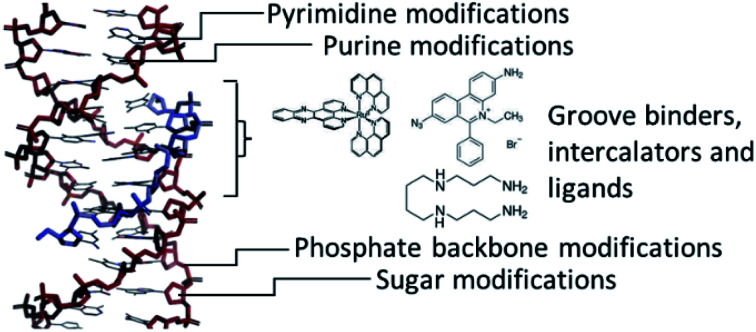

## 1. Introduction

DNA is the carrier of genetic information in all cellular systems and in many viruses. As the carrier of genetic material, it directs its own replication during the cell division process and the transcription of complementary molecules of RNA. One of the defining features of DNA is its structural flexibility. DNA can adopt a wide range of higher order structures including the duplex, G-quadruplex, i-motif and Holliday Junction, all of which either have a confirmed or suspected role in gene regulation and/or transcription processes^1^ and have been investigated in the context of ligand targeting.^2^

The DNA triplex is of particular interest due to its potential for exploitation in the targeting of therapeutics to specific DNA sequences. The triplex is formed when a DNA duplex is joined by a third strand, which binds in the major groove of the duplex to form a three-stranded assembly. Research efforts have increasingly focussed on TFO modification, to aid delivery *in vivo*, reduce or prevent degradation by nucleases and increase triplex stability. However, to fully understand the structure of the DNA triplex and how modifications and bound ligands can affect its stability, it is important to first examine the structure of the DNA duplex.

**Fig. 1:**
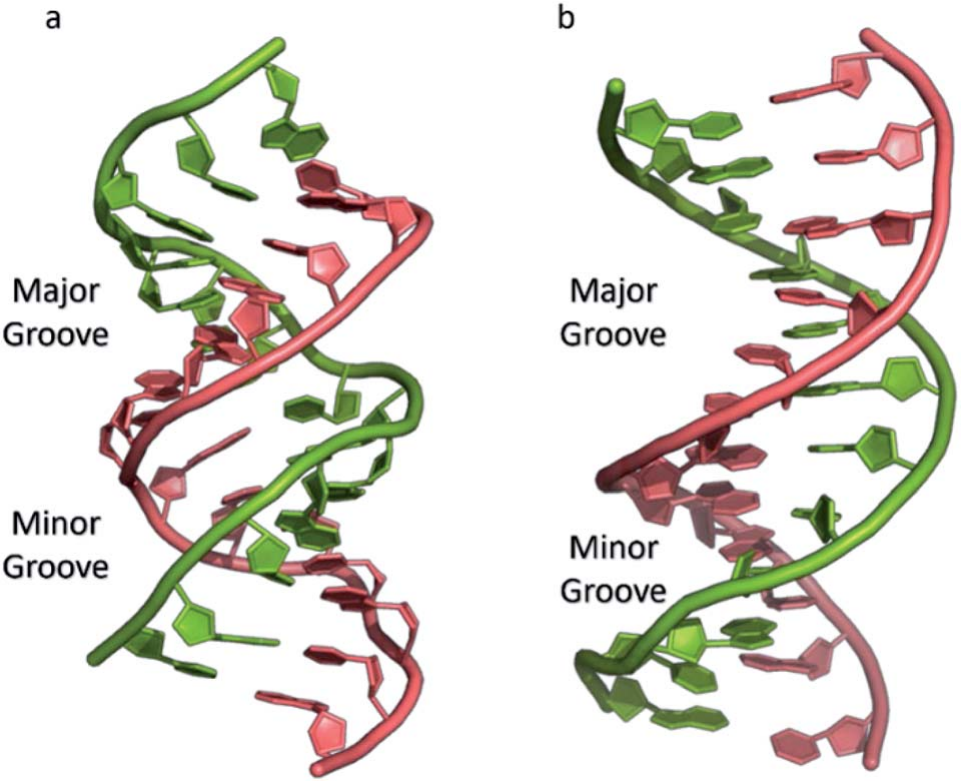
(a) A-DNA, (b) B-DNA.

The most common and best-known form of DNA is the B-DNA form, characterized by two polynucleotide strands with a right-handed helical twist about a long axis to form a double helix, bound together by hydrogen bonds and further stabilised by π-stacking between adjacent bases. This winding generates two grooves: the major one is wide and deep, while the minor groove is narrow (Fig. 1). This structure has been widely characterised by X-ray diffraction and occurs at high humidity and with a variety of DNA counterions including Na^+^, which serves to balance the negative charge of the phosphate backbone.^3,4^ The most significant characteristic of B-DNA is the possibility to accommodate only two types of naturally occurring base pairs (*i.e.*, adenine–thymine A–T and cytosine–guanine C–G). In B-DNA both base pairs can be replaced by each other without altering the position of the sugar–phosphate backbone, although runs of A–T base pairs are known to have a narrower minor groove. Similarly, the double helix is not disturbed by swapping the partners (*i.e.*, changing a C:G with a G:C or a T:A with a A:T). However, different combinations of bases lead to the formation of non-Watson–Crick base pairs with a significant distortion of the double helix. Since the variation of pairing causes distortions, DNA is a molecule able to adopt different non-canonical structures whilst exposed to physiological and non-physiological conditions. When the relative humidity is reduced to 75%, the B-DNA changes conformation, adopting the so-called A-DNA form, which presents a wider and flatter right-handed helix compared to the B-DNA form (Fig. 1). In contrast to the right-handed form, Z-DNA is a left-handed analogue which has a deep minor groove and a shallow but wide major groove.^5,6^ Z-DNA is formed as a function of DNA sequence and contains long sections of alternating purine–pyrimidine bases, most commonly as GC repeat units.

**Fig. 2:**
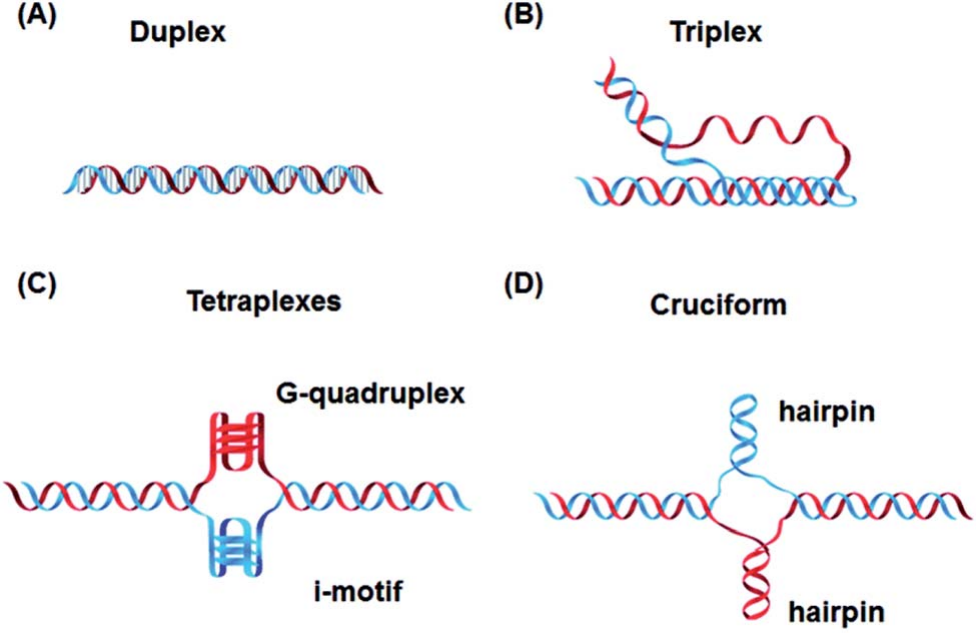
Canonical DNA structure and non-canonical structures including (A) duplex, (B) triplex, (C) G-quadruplex and i-motif and (D) hairpin. Reprinted from H. Tateishi-Karimata and N. Sugimoto, *Chem. Commun.*, 2020, **56**, 2379.

In addition to these, DNA can also form other non-canonical structures as a function of sequence, which are especially stable in the crowded intracellular environment. These arrangements were demonstrated to play a role in different biological processes such as replication, transcription, translation and reverse translation.^7^ Three strands of DNA can form a triplex structure, which was initially predicted to exist in 1953 by Pauling and co-workers and subsequently observed by Rich and co-workers after mixing poly U and poly A ribonucleotides in a 2 : 1 ratio.^8,9^ Triplex formation has been identified both *in vitro* and *in vivo*,^10^ as will be discussed in Section 5. Tetraplex structures, known as G-quadruplexes, have also been observed in G-rich strands. They are formed in sequences containing multiple guanine tracts within a G-rich sequence and are bound together by Hoogsteen hydrogen bonding.^11^ G-Quadruplexes have interestingly been observed in many different locations, correlated with genomic regions that play a functional role such as replication origin sites, telomeres and promoter regions.^12^ Another type of tetraplex structure is the intercalated motif (i-motif), formed between C-rich strands in acidic conditions. C-Rich sequences are found in telomeres, and in promoter regions of many human genes, indicating a probable role in biological processes.^13^ Finally, the cruciform structure is formed by intra-strand base pairing of inverted repeat sequences. It can be either a four-way junction or a three-way junction depending on the number of hairpins present (Fig. 2).^14^ In this review, we will focus on DNA triplex structures, discussing their structural characteristics, stability, and their potential applications. The DNA triplex has been investigated for decades as a very promising tool in gene editing, but development has been challenging, due both to the low thermal stability of the structure, and the poor cellular uptake of the triplex-forming oligonucleotides. The possible biological application of triplexes and approaches to mitigate their limitations will be covered in this review. The application and interaction of ruthenium polypyridyl complexes with DNA triplexes will also be discussed, to explore potential future therapeutic applications in areas such as photodynamic therapy (PDT). Ruthenium polypyridyl complexes possesses useful properties which are particularly suitable for biological applications, as presented in Section 6 of this review. Indeed, Ru-based compounds have been intensively studied in the last decades as antiparasitic, antimicrobial or anticancer drug candidates.^15,16^ In particular, ruthenium polypyridyl compounds have attracted much interest.^17^ Their ability to absorb light *via* a metal-to-ligand charge transfer (MLCT) process among other charge transfers have made them very interesting tools for photodynamic therapy (PDT).^18,19^ Therefore, we suggest that the intrinsic triplexes' sequence-specific binding properties combined with the phototoxicity of ruthenium derivatives can be exploited together to obtain breakthrough tools in gene editing technology.

## 2. Type of triplexes

**Fig. 3:**
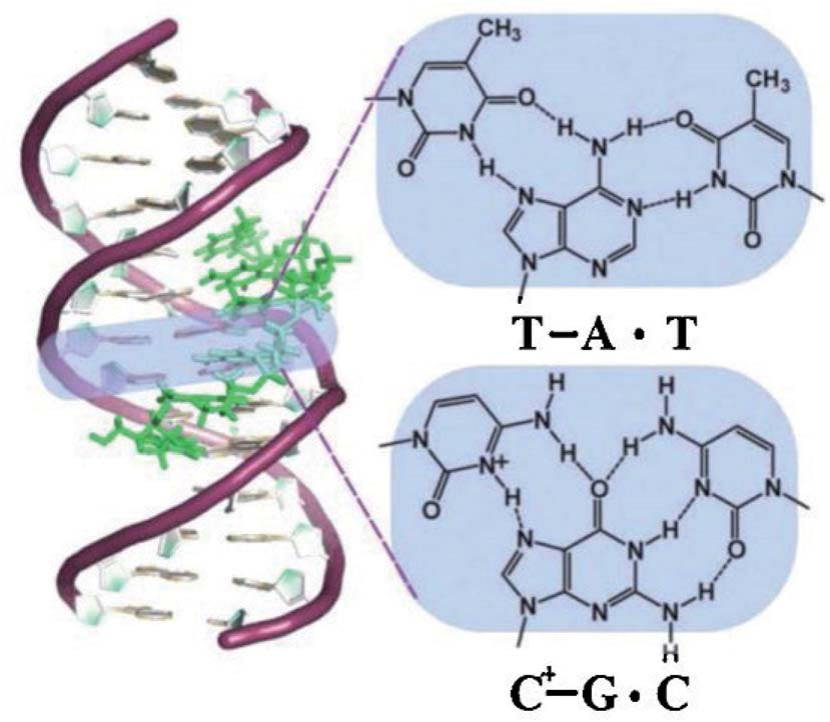
Schematic drawing of a triplex forming oligonucleotide that specifically recognises a DNA sequence, with the TFO binding in the major groove of the DNA duplex.^179^ “Reprinted from *Coord. Chem. Rev.*, **257**, Tarita Biver, Stabilisation of non-canonical structures of nucleic acids by metal ions and small molecules, 2765–2783, Copyright (2013), with permission from Elsevier.”

Triplex structures can be formed by DNA, RNA or hybrids of the two. This review focuses on DNA triplexes, so RNA-containing triplexes will not be considered here. DNA triplexes can be grouped based on the origin of the third strand. Intermolecular triplexes are formed between a double-stranded DNA (dsDNA) and an independent molecule termed the triplex-forming oligonucleotide (TFO). If the third strand is part of a single strand which also contains the dsDNA, the triplex is referred to as an intramolecular triplex. The hydrogen bonds between the two helices of DNA are typically Watson–Crick bonds, whereas the bonds between the duplex and TFO are either Hoogsteen or reverse-Hoogsteen bonds (Fig. 3). The directionality of the TFO can be either parallel or anti-parallel to the DNA strand which forms the hydrogen bonds.

### 2.1 Intermolecular DNA triplexes

**Fig. 4:**
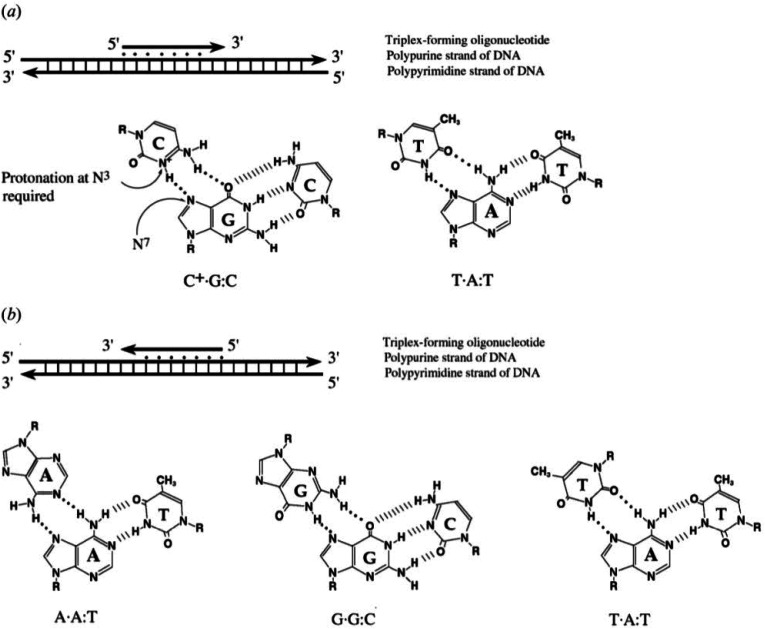
Intermolecular triplexes and canonical base triplets. (a) Polypyrimidine triplexes Y–R:Y (b) polypurine triplexes R–R:Y^96^ Reprinted from K. M. Vasquez and P. M. Glazer Triplex-forming oligonucleotides: principles and applications, *Q. Rev. Biophys.*, **35**, 89–107, copyright 2002, with permission from Cambridge University Press.

To explain the possible combinations of intermolecular DNA triplexes, a close examination of the sequence of the triplex-forming species is required (Fig. 4). In a polypyrimidine TFO that consists entirely of pyrimidines, the thymine will bind to the adenosine T–A:T or cytosine binds to guanine C–G:C, forming a triplex. The cytosine, however, requires a protonation of the N3 atom to ensure the second Hoogsteen bond with the guanine. Therefore, these parallel triplexes require a mildly acidic environment.^20^ However, there is a limit to protonation that, if not respected, will result in charge repulsion between the adjacent cytosines.^21^ When a TFO contains only purine bases, adenine binds to adenine (A–A:T) or guanine binds to guanine (G–G:C) with reverse-Hoogsteen hydrogen bonds. In contrast, a polypurine TFO forms a triple-helix by binding the duplex with an anti-parallel conformation.^22^ Additionally, in the anti-parallel conformation, it is also possible to have T–A:T steps within the DNA triplex.^23^

The base identity plays a key role in determining the local and overall twist angle of the DNA triplex. This residual twist is calculated based on the measurement of the angle between the two carbon atoms of the adjacent Hoogsteen base pairs and the base of interest.^24^ G–G:C triplets have the effect of increasing twist within the triplex, with an average increase of 10.6° per step, whilst T–A:T steps reduce the twist by the same value, with the overall twist angle of the helix being maintained at 30°. This is lower than the average twist for a B-DNA duplex of *ca.* 34° and therefore suggests that the binding of a TFO induces a slight unwinding of the duplex. This results in significant distortion after each A–T bond of the duplex within the polypurine triplexes. By contrast, the polypyrimidine triplex has much less backbone distortion and a higher number of hydrogen bonds between the TFO and the duplex, compared with polypurine. This reduction in distortion is one possible reason why parallel triplexes are generally more stable than antiparallel helices.^22^

### 2.2 Intramolecular DNA triplexes

In addition to the intermolecular DNA triplexes, where the TFO is an external oligo, the triplex can be formed by one DNA strand which folds back on itself, to form an intramolecular assembly. These are commonly referred to as H-DNA (hinged DNA), as their stability depends on the presence of acidic pH and negative superhelical stress. H-DNAs may be formed under supercoiled conditions with a mirror repeat polypurine–polypyrimidine sequence and the base motifs are the same as in the intermolecular triplexes with a pyrimidine third strand. Moreover, an intramolecular triplex composed with bases of pyrimidine–purine–purine in the DNA stretches, and a non-mirror repeat, is defined as *H-DNA.^25^

### 2.3 G-triplexes, R-DNA and PNA

It is also possible to form a triplex from G bases – the G-triplex, which contains a strand rich in guanine bases, and can be formed as an intermediate during the formation of a DNA G-quadruplex.^26,27^ Using fluorescence resonance energy transfer (FRET), it was determined that G-triplexes can assume both parallel and anti-parallel topologies.

A parallel DNA triplex may also be formed during homologous recombination and assists the recruitment of the homologous sequences. During the formation of the recombinant DNA (R-DNA), a complex with Rec-A may be formed, leading to a triplex with an extended rise distance of 5.1 Å, compared to a standard rise distance of 3.4 Å.^28^

Peptide nucleic acids, PNA, are modified oligonucleotides that contain a polyamide chain, instead of the sugar–phosphate backbone.^29^ Whilst the bases retain the canonical Watson–Crick pairing scheme, the PNA backbone lacks the negative charge associated with a phosphate backbone and therefore PNA can form a highly stable triplex with one or more DNA strands with reduced electrostatic repulsion. The binding directionality respect of the ds-DNA molecule can be both parallel, or anti-parallel forming a stable D-loop, *i.e.*, forming a momentary triple strand with one of the DNA strands.^30^ This DNA triplex can be seen as a triple-helix assembly, the stability of which can be increased by the incorporation of synthetic modifications, which will be discussed later in this article.

## 3. Structural analysis of triplexes

At time of writing, structural characterizations of triplexes are limited. Only 32 structures, with the majority solved using NMR, have been published in the Protein Data Bank.^31^ This includes triplexes composed of hybrids of DNA–RNA, DNA–PNA and RNA–RNA triplexes, some of which contain modified bases, sugars or intercalators, and excluding any structures which contain proteins. Only four of the structures published, containing DNA, have been determined using X-ray diffraction. DNA-containing triplex structures obtained by X-ray analysis were either formed with protein nucleic acid (PNA), intercalators, or as a result of DNA overlap with only a small number of bases forming Hoogsteen bonds and therefore do not represent a full and complete true DNA triplex, unlike several of the structures solved using NMR. Structure determinations of DNA-containing triplexes are summarized in Table 1.

**Table 1:** DNA-containing structures of triplexes deposited in the Protein Data Bank^31^

Intramolecular or intermolecular	Triplex type	Nucleic acid type	Method	PDB ID	Year	Reference
**DNA only**						
Intramolecular	Antiparallel	DNA	NMR	134D	1993	34
Intramolecular	Antiparallel	DNA	NMR	135D	1993	34
Intramolecular	Antiparallel	DNA	NMR	136D	1993	34
Intramolecular	Antiparallel	DNA	NMR	177D	1994	35
Intermolecular	Parallel	DNA	NMR	149D	1994	34
Intermolecular	Parallel	DNA	X-Ray diffraction	208D	1995	36
Intramolecular	Parallel	DNA	NMR	1AT4	1997	37
Intramolecular	Parallel	DNA	NMR	1D3X	1998	38
Intramolecular	Parallel	DNA	NMR	1BCB	1998	39
Intramolecular	Parallel	DNA	NMR	1BCE	1998	39
Intermolecular	Parallel	DNA	X-Ray diffraction	1D3R	1999	40
Intermolecular	Parallel	DNA	NMR	1BWG	1999	41
Intramolecular	H-DNA H-Y5 isomer	DNA	NMR	1B4Y	1999	42
Intermolecular	G-Triplex	DNA	NMR	2MKM	2014	43
Intermolecular	G-Triplex	DNA	NMR	2MKO	2014	43

**Modified DNAs**						
Intermolecular	P-Form	DNA + PNA	X-Ray diffraction	1PNN	1995	44
Intramolecular	Parallel	DNA + 1-(2-deoxy-beta-d-ribofuranosyl)-4-(3-benzamido)phenylimidazole	NMR	1WAN	1996	45
Intramolecular	Parallel	DNA + N7-glycosylated guanine	NMR	1GN7	1997	46
Intramolecular	Parallel	DNA + 1-propynyl deoxyuridine in third strand		1P3X	1998	47
Intramolecular	Parallel	DNA + LNA	NMR	1W86	2004	48
Intermolecular	P-Form	PNA	X-Ray diffraction	1XJ9	2005	49
Intramolecular	Antiparallel	DNA + TINA intercalator	NMR	6QHI	2019	50

**Fig. 5:**
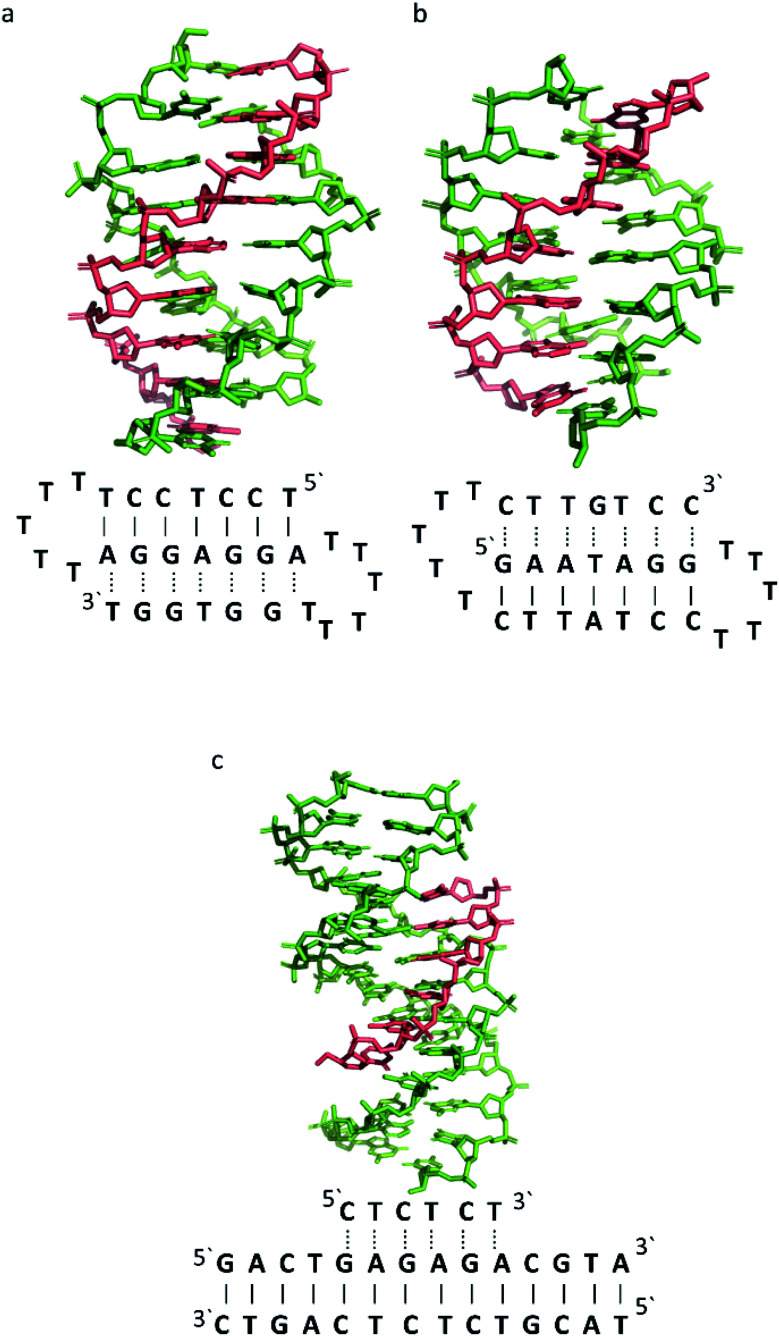
3D Representation of and schematic diagram of (a) triplex A (intramolecular antiparallel, PDB ID 134D), (b) triplex B (intramolecular parallel, PDB ID 149D) (c) triplex C (intermolecular parallel, PDB ID 1BWG). The TFO is displayed in red and the DNA duplex is in green. In the schematic diagrams, Watson–Crick hydrogen bonding is displayed using lines with Hoogsteen bonds illustrated in dashed lines.

To better illustrate the structural influence of the binding of a TFO to a DNA duplex to yield a triplex, three DNA triplexes, triplex A, B and C, were selected. The three structures were chosen as examples of triple helix structures which did not contain intercalators, other small molecules or chemical modifications. As the structures were solved using NMR, they are representative of triplex species in solution (Fig. 5).

### 3.1 Similarity with B-DNA

B-DNA is a right-handed form of the double helix, with 10.1 base pairs in each turn^3^ and a helix diameter of 20 Å.^32^ Fibre diffraction data, obtained from X-ray studies, show that the average value of the helical twist per base pair is 36.1°, but that this can vary from 24° to 51°. The distance between bases (rise) is 3.4 Å per base pair. Whilst B-DNA is the most frequently encountered DNA conformation in physiological conditions,^33^ others are possible, including A- and Z-forms, and are promoted by both sequence and changes in the DNA microenvironment.

The B-DNA structure forms two grooves, a minor and major with a width of ∼5.7 Å and ∼11.7 Å, respectively. The value is obtained by subtracting 5.8 Å from the distance between the phosphate groups on opposing strands, which is the van der Waals radius of one phosphate group.^51^

The DNA triplex possesses significant similarity in structure to the B-form duplex. The base rise distance remains consistent at 3.3 Å and the twist value of triplex A is also similar to a standard B-form duplex at *ca.* 34°. Triplex A contains a poly-purine TFO, as illustrated in Fig. 5.

**Fig. 6:**
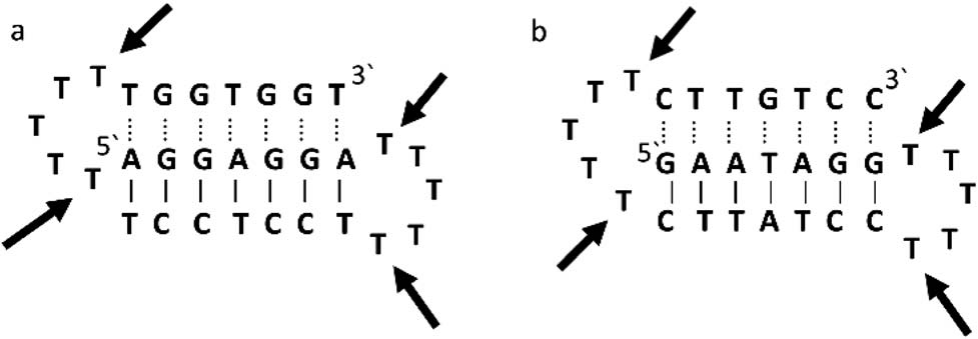
Schematic representation of (a) triplexes A (PDB ID 134D) and (b) triplex B (PDB ID 149D). The arrows indicate the four thymine that are reported in the analysis, but do not bind to any complementary base.

Triplex A is an intramolecular triplex constructed from a single oligonucleotide. However, the loop positions could not be assigned due to disorder and are therefore not included in the structural coordinates. Whilst loop bases may not form hydrogen bonds with each other or with the TFO, and therefore disorder within this region is expected, the T bases indicated by arrows in Fig. 6 adopt T–T wobble pairs, indicating two hydrogen bonds are present between the first T bases in each loop. The average base pair twist at this pair is 32° which is slightly reduced compared to the average helical twist value for B-DNA (36.1°). However, other than this there is no significant perturbation to the duplex part of the triplex structure compared to B-DNA, highlighting that the interaction of the TFO-region in the major groove does not significantly alter the structure of the template duplex.

**Fig. 7:**
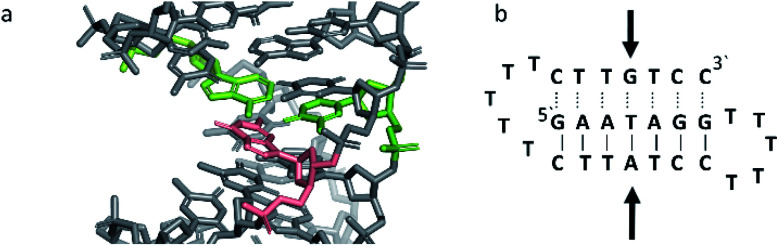
(a) 3D and (b) schematic representations of the G–T:A triplet of the triplex B. Green indicate the duplex bases, guanine and adenine, while the orange base is the guanine of the TFO.

Triplex B is also an intramolecular triplex but with a TFO composed of purine bases that bind the duplex strand in a parallel arrangement (Fig. 5). Whilst the overall structure shows little difference with that of triplex A, which adopts an antiparallel arrangement, local distortions can be observed in individual base triplets. The most significant of these is in the central step within the triplex, as indicated by arrows in Fig. 7b. At this step, the G base in the triplex strand is unable to form a proper binding interaction with the T–A base pair (a T base would be needed for this to occur), as illustrated in Fig. 7. Whilst this mismatch of bases would be expected to reduce the overall stability of the triplex assembly, individual sites of mismatched bases do not necessarily prevent triplex formation.

Perturbation of other derived parameters within the structure, including changes in the propeller and buckle value either side of the mismatch site, indicate that this single step of instability may result in an overall reduction of stability or rigidity across the triplex assembly^6^ despite an overall twist value of 30.8°, which is reduced compared to that found for B-DNA.

In triplex C the TFO is a purine-rich hexamer oligonucleotide that binds in the major groove of a 13 base-pair duplex, forming a parallel triplex assembly (Fig. 5). In contrast to triplexes A and B, triplex C is an intermolecular assembly and the length of the TFO is shorter than the duplex to which it is bound. As illustrated in the schematic representation of the structure (Fig. 5), the TFO forms both TA × T and CG × C+ triplets, with charge neutralization of the C+ bases by the phosphate backbone being expected to confer greater stability on the assembly.^41^

Whilst the triplex section of triplex C is structurally similar to A and B, this structure gives insight into the structure of the helix either side of the TFO. Whilst the overall twist angle per step within the triplex region is maintained at *ca.* 33, the remaining duplex steps display much greater variability, with twist angles ranging from 29–45° per step.

The reduced twist angle common to triplex structures raises the question of whether the duplex component is closer to B-DNA or A-DNA in conformation, the latter of which is characterized by a reduced twist of *ca.* 32° per base in combination with a dominant C3′-*endo* sugar pucker for the ribose ring in the bases.

**Fig. 8:**
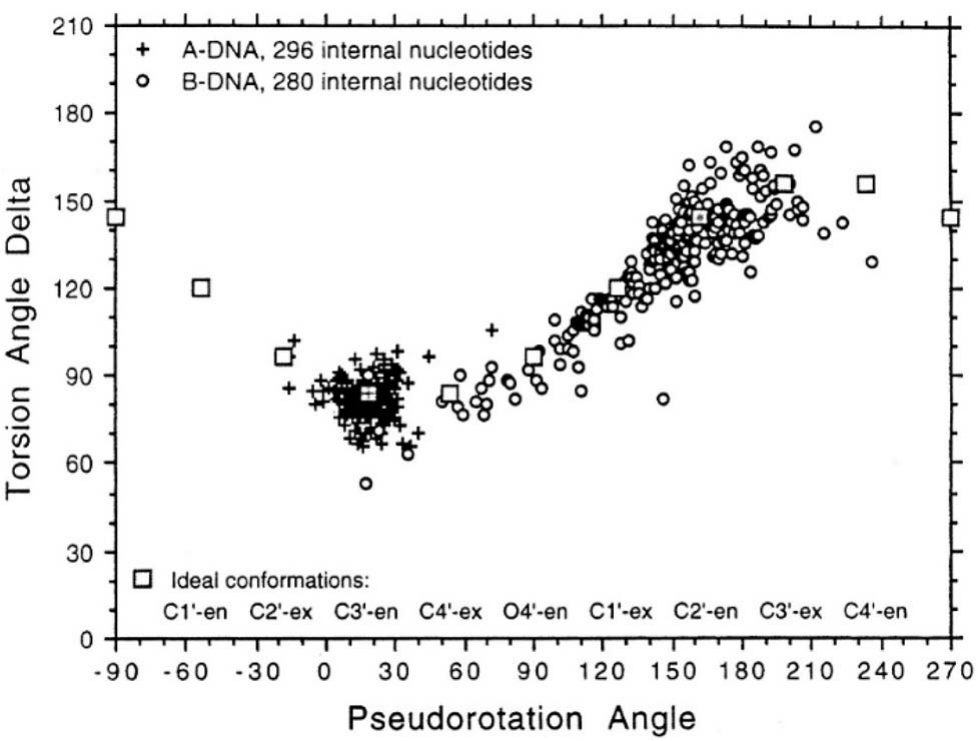
Representation of sugar rings of B-DNA (circle) and A-DNA (crosses) based on pseudorotation and torsion angle.^180^ Reproduced with permission from R. E. Dickerson, International Tables for X-ray Crystallography, Volume F: Macromolecular Crystallography, ed. M. G. Rossmann, E. Arnold (International Union of Crystallography, Chester, U.K. (2001).

To determine whether the DNA triplex has an A or B conformation, the angle values needed are the backbone sugar torsion *δ*, the glycosyl torsion *χ* or the pseudorotation angle of sugar rings P⋯P.^52^ Typically, the duplex can adopt the A- or B- form, and this is dependent on the sugar pucker adopted in each nucleotide. An A-form is adopted when the dominant sugar pucker is C3′-*endo*, with a pseudorotation value of between −30° and 40°, while a wider range of pseudorotation values can be indicative of the B-DNA conformation. Indeed, the B-conformation is not limited to the C2′-*endo* pucker, where the majority of the nucleotides can be found, but can adopt several other forms including C4′-*exo*, O4′-*endo*, C1′-*exo*, C3′-*exo* and C4′-*endo*^53^ (Fig. 8). The dominant sugar pucker can be used to assign the overall conformation of the helix and is particularly important for the development of ligands designed to target specific steps, as a change in sugar pucker will change the spatial arrangement of atoms around the binding site, potentially changing the mode of interaction by the ligand. The overall conformation of the duplex component of the triplex can be assigned to a conformation using the pseudorotation value (*P*) for each base.^54^

In triplex A, the *P* value for the bases forming the duplex lie within the range of 100–160°, indicating a majority B-DNA conformation. Whilst the terminal bases in the duplex lie outside of this range, this could be because of torsional stress placed on the structure due to the folding of the loops, which have not been presented in the coordinates for the structure. The TFO strand however, displays much less variation in the sugar pucker values. Whilst these again indicate a B-like conformation, the majority of values are either *ca.* 176° or are within a range of 50–70°, indicating less flexibility in the TFO strand compared to the duplex. This trend, of an overall B-DNA conformation for the duplex matched with less variation in the P for the TFO, is observed in all three structures. However, there are exceptions such as the central G:T–A triplet in Triplex B, which has a (*P*) value of 19.0°, indicating an A-like C3′-*endo* sugar pucker. This may be a pucker which is sequence dependent, or could indicate torsional strain in the TFO which is corrected in the central step by the adoption of this unusual pucker.

Due to the relatively small number of DNA triplex structures available, it is not yet possible to identify the expected structural variation as a function of sequence. However, a better understanding of the structural variation expected for the DNA triplex may assist with the development of ligands designed to bind to specific sites within the assembly, to understand the distinctive behavior of triple helices more structural analysis is essential.

## 4. Stability of DNA triplexes

DNA triplexes are inherently less stable than their duplex counterparts in part due to the increased negative charge density from the phosphate backbones, which increases repulsion between the strands. However, multiple factors can affect the stability of a triplex assembly including the presence and concentration of monovalent or divalent cations, pH and temperature. Additionally, triplex hybridization can be promoted by the presence of molecular crowding and chromatin accessibility in the biological environment.

Efforts have been made to increase triplex stability through chemical modification of the base, sugar, or phosphate backbone of DNA.^55,56^ Base modifications have been the focus of extensive synthetic efforts due to pH being one significant factor which can negatively affect triplex stability. Modification has also focused on changing the phosphate and sugar within the nucleotides to enhance resistance to nucleases in the cell, in order to reduce degradation, and to enhance the ability of the TFO to enter and bind in the major groove of the duplex.

Finally, the use of ligands, such as intercalators or groove binders, has been explored as one approach to increase triplex stability without chemical modification to the TFO, although this is a secondary effect of targeting the triplex assembly with such a molecule.

### 4.1 Cations and anion enhancement of DNA triplex stability

The cellular microenvironment exercises direct control over triplex stability and activity at the molecular level. Considering the intense negative charge of a structure that is formed by three strands of DNA, a high concentration of multivalent cations will mitigate the repulsion.^57^ Generally, it is agreed that the formation of intermolecular triplexes with a polypurine sequence requires divalent cations^58^ such as Mg^2+^, whereas for the intramolecular assembly, sodium ions are sufficient. It has also been demonstrated that the inclusion of Mg^2+^ can contribute to an increase in stability of reverse Hoogsteen bonds, resulting in an increased thermal stability for intramolecular triplexes.^59^

Several cations can increase triplex stability. For divalent cations, the order of stabilisation is Mg^2+^ > Mn^2+^ > Ca^2+^ > Ba^2+^, which can be attributed to the ionic radius of each ion – the smaller the radius is, the greater the alignment between nucleotides and hence the greater the stability of the triplex assembly.^60^

In contrast, monovalent ions, such as the physiological concentration of K^+^, reduce the propensity of a G-rich strand to form a triplex. The presence of molecular crowding conditions (which are often simulated *in vitro* by using high concentrations of polyethylene glycol, such as PEG 200) can also affect the formation of triplexes, and with a G-rich strand in the presence of Ca^2+^, the formation of a G-triplex is promoted with endothermic energy.^61^ Molecular crowding conditions can also promote triplex formation and change the effect on stability of adding monovalent ions. For example, in the absence of crowding conditions the addition of K^+^ has been demonstrated to increase triplex stability as a function of K^+^ concentration. However, in crowding conditions the addition of K^+^ actually reduces the stability of the triplex assembly.^62^

Using a crowding agent along with ions to simulate the environment in which triplexes might be found, short triplexes tend to stack together and form a highly condensed structure.^63^ Since this effect was also observed with duplexes, it has been argued that DNA triplexes may affect the genome structure with modification at a chromosome level.^64^

### 4.2 Base modifications

An increasing number of oligonucleotide analogues have been developed to obtain TFOs with increased stability (both of the resulting triplex and increased resistance to degradation by nucleases) and enable greater selectivity of targeting towards specific structures or DNA sequences.^55,65^

#### 4.2.1 Base modifications in parallel triplexes

Parallel triplex stability can be increased when the sequence contains a greater number of C^+^–G:G triads rather than T–A:T steps, but the observed stability is still pH-dependent, with an optimal pH below 6.2.^66^ Indeed, the protonation of the cytosine bases will provide a second hydrogen bond between the N-3 of cytosine itself and the N-7 of guanine, favouring a Hoogsteen bond and consequently the triplex formation in mildly acidic conditions.^67^

**Fig. 9:**
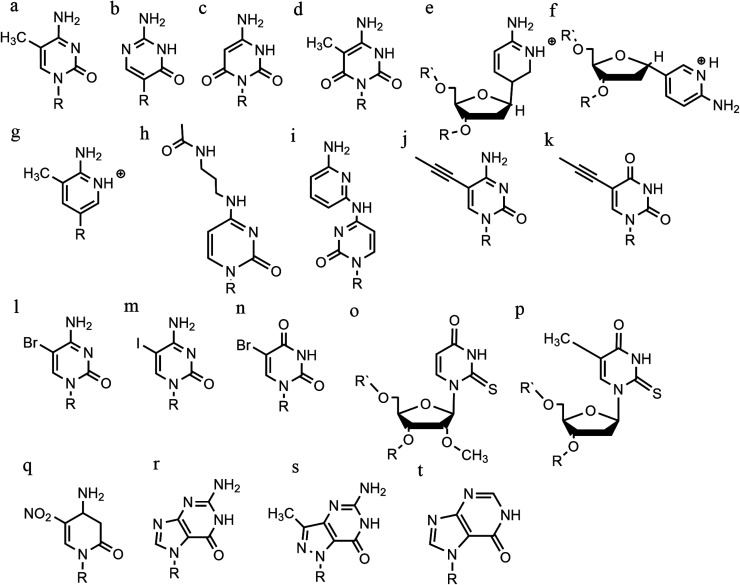
Base modifications in parallel triplexes. (a) 5-Methyl-cytosine, (b) 2′-*O*-methyl-pseudoisocytidine, (c) 6-oxo-cytosine, (d) 5-methyl-6-oxo-cytosine, (e) α-AP, (f) β-AP, (g) 2′-aminoethoxy-thymine, (h) *N*^4^-3-acetamidopropyl-cytosine, (i) *N*^4^-6-aminopyridinyl-cytosine, (j) 5-propynyl-cytosine, (k) 5-propynyl-uracil, (l) 5-bromo-cytosine, (m) 5-iodo-cytosine, (n) 5-bromo-uridine, (o) 2′-*O*-methyl-2-thio-uridine, (p) 2-thio-thymidine, (q) 6-amino-5-nitropyridin-2-one, (r) N7-glycosilated-guanine, (s) P1-guanine, (t) inosine.

However, a series of C bases in a tract will result in lower triplex stability, due to the proximity of multiple charges from the protonated bases, which require more acidic conditions to stabilise.^21^ This has prompted researchers to focus on cytosine analogues that support pH-independent triplex formation *e.g.*, neutral cytosines with two hydrogen donor groups, or analogues that protonate more easily. To reduce pH-dependency, modifications to cytosine have been explored, with the aim of increasing triplex stability in a wider pH range. The methylation of cytosine in the TFO, in 5-methyl-cytosine, contributes to the base stacking, increasing stability (Fig. 9a).^55,66^ In recent calculations, it was demonstrated that the methylation of C also shifts the pKa from 4.6 (for cytidine) to 4.9.^68^ The 2′-*O*-methyl-pseudoisocytidine (Fig. 9b) promotes the formation of triplexes in a neutral environment in a TFO that will recognize GC-tracts, exemplified using poly(GC). However, this modification is not widely used because the synthesis is highly challenging, even though it is an excellent candidate for increasing the stability of parallel triplexes. The stabilising effect of this pseudo-isocytosine is reported in intramolecular triplexes with a loop composed of only two bases.^69^ The incorporation of 6-oxo-cytosine can increase triplex stability to above pH 7, however, when compared with triplexes containing protonated or methylated cytosines in more acidic conditions, the stability decreases (Fig. 9c). The analogue 6-oxo-cytosine can be further modified by the addition of a methyl group in position 5, obtaining 5-methyl-6-oxo-cytosine (Fig. 9d), which can also promote the stability of the DNA triplex. The use of glycerol linkers combined with 6-oxocytosine has been proposed as a modification, which reduces the steric interaction between the 6-carbonyl and the sugar, increasing the stability of the triplex in comparison to the stability observed with no linker present. The absence of glycerol linkers particularly reduces the stability of the triplex if it contains a G-tract.^70^ The incorporation of 2-aminopyrimidine (AP) can promote increased triplex stability at physiological pH without protonation due to the low basicity of the modified base. AP can be incorporated in the TFO as β and α-anomers, the first cytosine anomer has a lower pH dependency due to its p*K*_a_ of 6.5, resulting in stable triplexes (Fig. 9e and f).^71,72^ Unsurprisingly, the addition of a 5-methyl-cytosine in the same TFO containing the β-AP does not form triplexes because of the unfavourable steric interaction. An alternative is a combination of the methylated version of the 2-aminopyrimidine with the 2′-aminoethoxy-thymine (Fig. 9g) reaching a binding affinity at pH 9.0.^73^

In terms of base recognition, modified oligonucleotides play a crucial role in enhancing sequence-specific recognition. In the case of parallel triplexes, a TFO with *N*^4^-3-acetamidopropyl-cytosine can recognise a GC base pair, by forming a more stable triplex due to the increased chain flexibility (Fig. 9h) and has higher stability than the equivalent TFO containing only cytosine.^74^ A similar example reported is *N*^4^-6-aminopyridinyl-cytosine, which can recognise pyrimidine base interruptions in a polypurine sequence (Fig. 9i).^75^ The addition of a propynyl group can increase triplex hydrophobicity and consequently stacking interaction. Another example reported is 5-propynyl-cytosine, which replaced the cytosine, but when the propynyl group was attached to uracil, the TFO with 5-propynyl-uracil is more favourable for the stability of parallel triplex compared to the 5-propynyl-cytosine (Fig. 9j and k).^55,76^

Cytosine analogues containing bromine or iodine atoms at position 5 have also been explored, obtaining 5-bromo-cytosine and 5-iodo-cytosine respectively, but the incorporation of these into a TFO, by replacement of cytosine, actually reduced triplex stability (Fig. 9l and m). Instead, the substitution of thymine by a 5-bromo-uridine (Fig. 9n) enabled the formation of triplex at room temperature. The inability to obtain triplex structures with 5-halocytosine derivatives is explained by their lower p*K*_a_ and the requirement of protonation.^77^ Some studies show that the use of modification on both uracil and thymine in a TFO, such as 2′-*O*-methyl-2-thio-uridine and 2-thio-thymidine increase the stability of a DNA parallel triplex and the reason is the stacking properties of the 2-thiocarbonyl on the 5′ of the upper thiouracil base and the nitrogen atom of the 3′ of the lower pyrimidine (Fig. 9o and p). Additionally, it is emphasized that a TFO that includes thiocarbonyl moieties recognizes a base mismatch, a key feature for antibody therapies.^78^ A recently published study proposed a TFO containing 6-amino-5-nitropyridin-2-one that overcomes the need for protonation, by acting as an uncharged mimic which can form a parallel triplex, with *in vitro* evidence demonstrating that this approach shows promise (Fig. 9q). The modified nucleobase was included in the TFO through an enzymatic process at physiological pH, relying on the thermodynamic stability of 6-amino-5-nitropyridin-2-one compared to other mismatched bases. Additionally, the modified TFO enhanced protection to the DNA from nucleases.^79^ The modification of purine bases has also been explored, although this has received less attention than the pyrimidines. A substitution of N7-glycosylated-guanine or P1-guanine with a cytosine has a remarkable impact on the triplex stability when in the presence of a G-tract (Fig. 9r and s).^46,80,81^ If guanine is converted into inosine by removal of the guanine-*N*^2^ amino group, then this is able to recognise a GC base pair and form a triplex structure. Additionally, the absence of the amino function give space to an unusual bond between the carbonyl group of the modified base and the CH of the guanine of the duplex, resulting in a higher electrostatic stability (Fig. 9t).^82^

#### 4.2.2 Base modification in anti-parallel triplexes

**Fig. 10:**
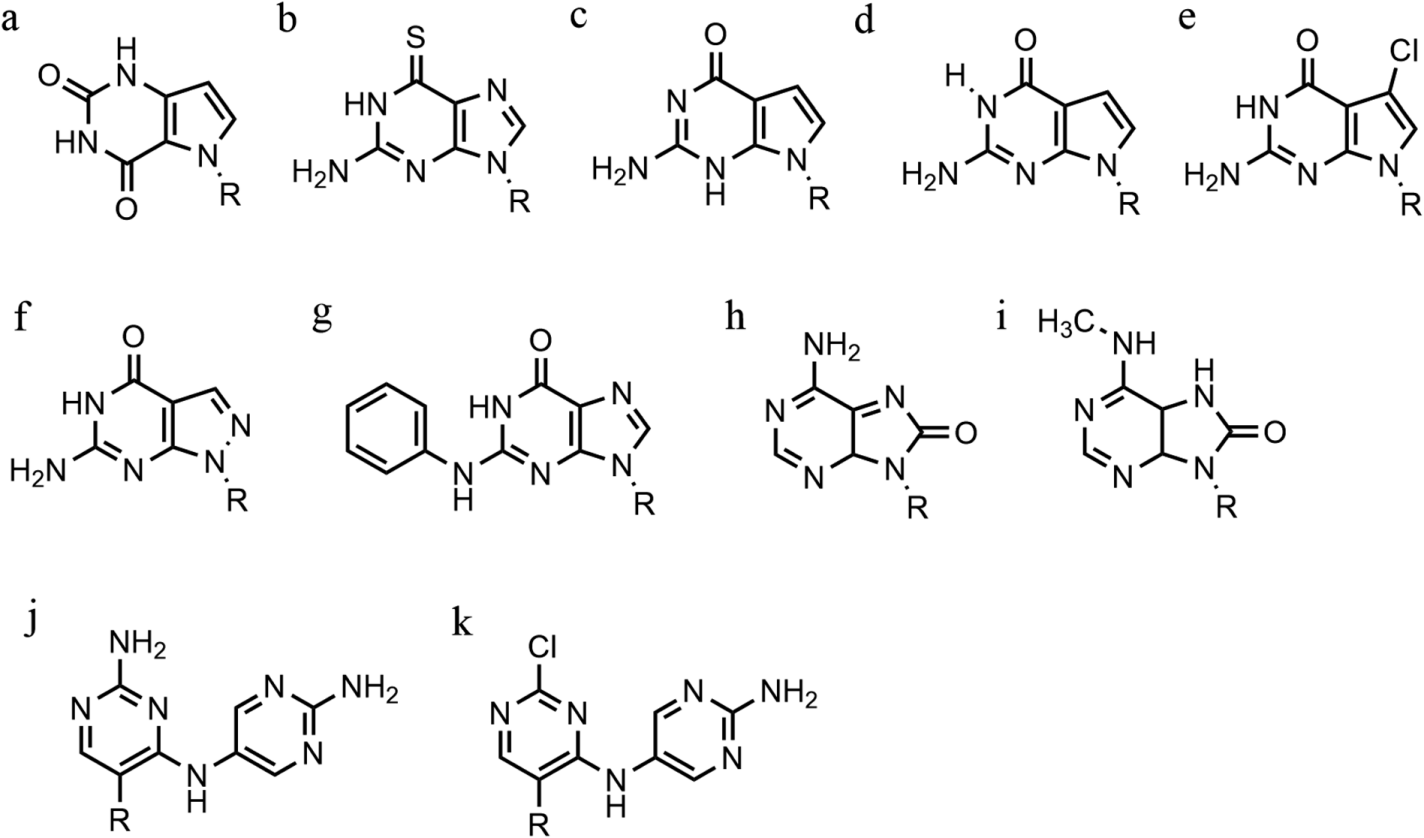
Base modification for anti-parallel triplexes. (a) 7-deaza-xanthine, (b) 6-thioguanine, (c) 9-deaza-guanine, (d) 7-deaza-guanine, (e) 7-chloro-7deaza-guanine, (f) 8-aza-7-deaza-guanine, (g) PhdG, (h) 8-oxo-adenine, (i) *N*^6^-methyl-8-oxo-adenine, (j) AY-d(Y-NH_2_), (k) AY-d(Y-Cl).

The principal concern when working with anti-parallel triplexes is the competitive formation of a G-quadruplex structure due to large numbers of guanine residues. It has been reported that the physiological level of K^+^ (over 100 mM) will stabilize the formation of quadruplexes rather than triplexes. Therefore, the aim of chemical modification is to produce analogues which will prevent quadruplex formation whilst promoting the formation of a parallel triplex. To stabilise an antiparallel DNA triplex, it was proposed to replace thymine with a 7-deaza-xanthosine (Fig. 10a). The introduction of this modification will reduce the likelihood of the oligo assuming a G-quadruplex structure, since the N7 needed as a hydrogen donor in the modified guanine is absent.^83^ The essential role of potassium suggests, 6-thio-guanine should prevent the formation of quadruplexes due to the very weak electron pair donor properties of the S lone pairs to K^+^ ions, compared to the carbonyl group (Fig. 10b).^84,85^ Other examples of analogues that prevent K^+^ coordination are 9-deaza-guanine, 7-deaza-guanine and 7-chloro-7deaza-guanine, and although they will form triple helical structures, there is no sign of significantly increased triplex stability (Fig. 10c–e).^86–88^ Instead, a purine modification in parallel triplexes that can form triplexes in G-rich TFO at physiological [K^+^] is 8-aza-7-deaza-guanine (PPG) (Fig. 10f).

Furthermore, modified TFOs containing this modification were used in cells to generate triplex-induced mutations and to cause double-strand breaks (DSBs) that will lead to cell death. These results show the modification forming a stable triplex, preventing G-quartet formation and inducing gene modification, editing and cell apoptosis.^89^ Another, more recent, modification included in antiparallel triplex DNA is the product from a synthesis of a guanine derivative *N*^2^-phenyl-2′-deoxyguanosine (PhdG), which was shown to form a stable and selective triplex with the GC base pair (Fig. 10g). As a drawback, as more PhdG bases are introduced, there is an increased likelihood that the structure could assume a higher order.^90^ Finally, to support the triplex formation in the presence of high [K^+^], the protonation of the backbone is often used as an alternative approach,^91^ which will be discussed in the next section.

The modification of adenine has also been reported as a potential route to enhancing triplex stability. The purine analogue 8-oxo-adenine forms stable Hoogsteen bonds with a G:C Watson–Crick base pairing (Fig. 10h). Additionally, an *N*^6^-methyl-8-oxo-adenine binds a purine sequence improving the triple helical stability (Fig. 10i).^92^ The 8-NH_2_ modification of the 8-amino-purine creates a stable interaction either with cytosine or guanine. Therefore, numerous 8-amino-purine derivates were tested in DNA triplexes, demonstrating that, regardless of structural alterations to the chemical structure, antiparallel triplexes are found to be more stable in physiological pH conditions.^93^

Pyrimidine derivatives have been exploited to stabilize anti-parallel triplexes. The incorporation of a cytosine nucleoside containing an amino-pyrimidine unit AY-d(Y-NH_2_) or AY-d(Y-Cl), results in stable triplexes able to recognise the inverted G:C instead of the canonical C:G, or T:A instead A:T with a duplex (Fig. 10j and k).^94^

### 4.3 Phosphate backbone modification

An alternative strategy to promote triplex stability is to focus on modifications to the phosphate backbone of the oligonucleotide.^95^ In general, TFOs are more likely to form a self-associated structure when the backbone is neutral or cationic, due to a decrease of the electrostatic repulsion between the three anionic strands. Modifications have been designed to promote a higher affinity between the TFO and the duplex strands whilst also increasing TFO nuclease resistance, which is important for the longevity of a TFO strand inside a cellular environment.

**Fig. 11:**
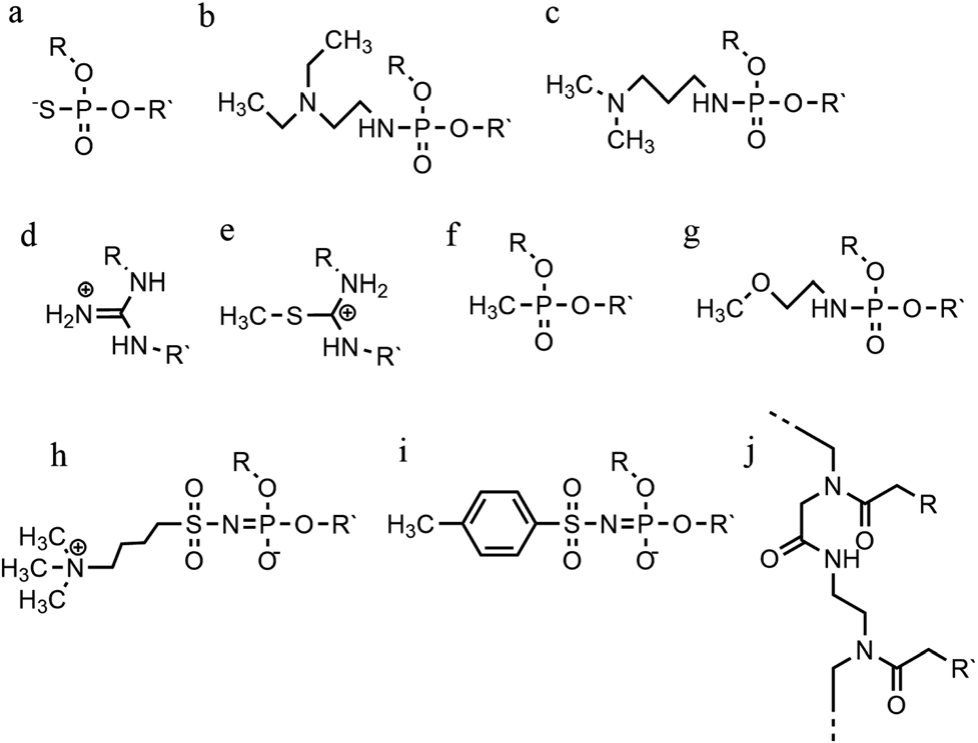
Phosphate backbone modifications. (a) Phosphorothioates, (b) DEED, (c) DMAP, (d) guanidino, (e) methylthiourea, (f) methyl-phosphonates, (g) PNHME, (h) azido-phosphoramidate, (i) tosyl sulfonyl phosphoramidite, (j) PNA.

A significant number of backbone modifications have been explored in the context of DNA triplexes. One of the first to be produced, the phosphorothioate modification (S-oligos), included a substitution to one of the non-bridging oxygen atoms in the phosphate group, replacing the O with S. This modification presents a significant drawback when applied *in vivo*, as TFOs containing this modification tend to bind proteins non-specifically. So, whilst this modification does confer nuclease resistance to the TFO, increasing longevity in the cell, it still maintains the negatively charged backbone, which is thought to reduce triplex stability (Fig. 11a).^96^

The formation of positively charged backbones have been explored, with the incorporation of cationic amine groups into the DNA backbone, including groups such as *N*,*N*-diethyl-ethylenediamine (DEED) or *N*,*N*-dimethyl-aminopropylamine (DMAP) (Fig. 11b and c). These modifications increase the binding affinity of the TFO *in vitro* and make them increasingly nuclease-resistant.^96^ Increasingly complex changes in the backbone modification have been also proposed. Guanidino and methylthiourea are some examples of a complete substitution of the phosphate group with a cationic linked nucleoside, resulting in more stable triplex oligomers even though an increasing proportion of T and A in parallel triplexes decreases the melting temperature (Fig. 11d and e).^97,98^

Options to obtain non-ionic alternatives are available as well, such as methyl-phosphonates, phosphotriesters and non-phosphate hydrazide derivatives (Fig. 11f). However, the lack of charge makes them highly insoluble, and they are therefore less suited to *in vivo* applications.

Different phosphoramidate-linkage modified TFOs have been proposed to bind the dsDNA efficiently by enhancing DNA stability.^99^ An example of a phosphoramidate-modified oligonucleotide is methoxyethylphosphoramidate (PNHME) for pyrimidine with the α-anomeric configuration (Fig. 11g).^100^ Whilst backbone repulsion is decreased, the triplex is only formed at pH 7 or lower and therefore this process is still protonation dependent.

Recently, an increasing number of studies have incorporated zwitterionic modifications in oligonucleotides, yielding thermostable triplexes. For example, an azidosulfonyl ammonium salt can be used, instead of TsN_3_, to give a zwitterionic derivative (see Fig. 11h). As a consequence, the duplex formation is less dependent on ionic strength. While this change increased the hydrophobicity of the molecule compared to the unmodified DNA, stable parallel triplexes form at a pH optimum of 5, and only when the modification is at the 3′ end. Furthermore, the presence of a tosylsulfonyl phosphoramidite (Ts) can be exploited as a negatively charged phosphate (Fig. 11i). Both modifications, when introduced into the same oligonucleotide, form stable parallel DNA triplexes and show promise for *in vivo* applications, especially as nuclease resistance and cellular uptake were increased compared to non-modified oligonucleotides.^101^

A more drastic modification of the phosphodeoxyribose backbone features the use of peptide nucleic acids, PNA (Fig. 11j). A PNA strand was conceived as a triplex-forming oligonucleotide, able to bind to a dsDNA due to its neutral charge. Subsequently, it appeared that two PNA strands, where the phosphate backbone is substituted by units of *N*-(2-aminoethyl) glycine, form remarkably stable triplexes when binding the unmodified TFO. The high stability of the triple-helical structure arises primarily from the neutral charge, drastically reducing backbone repulsion. Molecular dynamics simulations confirm that PNA backbones provide additional flexibility to the triplex and in some cases can assume A-type conformations.^102,103^ An alteration of PNA was proposed with an arginine instead of glycine, forming the G-PNA. This modification has overcome the solubility issue of PNA.^104^ Two other modifications reported are olefinic peptide nucleic acids (OPA) and oxy-PNA. These alternatives seek to improve the cellular uptake rather than the triplex stability itself.^105,106^ Alternatively, PNA can contain ligands with coordinated metal ions instead of nucleobases. The outcome is a stable triplex in solution experiments, due to the strength of coordinative bonds compared to hydrogen bonds, but which is reduced by the steric interactions of the metallo-complex and the triplexes.^107^ The use of PNA has been explored in a number of different areas including cellular uptake,^108^ regulation of gene expression,^109^ interruption of the RNA polymerase and inhibition of translation and activation of DNA repair system.^95^ It shows great potential as a future therapeutic, and work in this area is ongoing to address some of the challenges associated with its use, such as cellular delivery.

### 4.4 Sugar modification

**Fig. 12:**
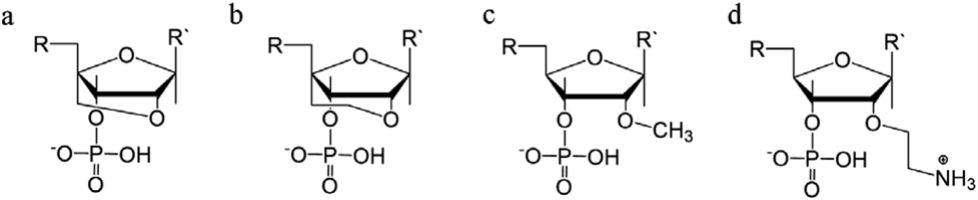
Sugar backbone modifications. (a) LNA, (b) ENA, (c) 2′-OMe, (d) 2′-AE.

Sugar modifications focus on the sugar pucker conformations that will influence the ability of the TFO to form a stable structure. The most common approach used to increase the stability of the triplex restricts the range of sugar conformations, relying on the use of bridged nucleic acids (BNA).^110^ The puckering characteristics of the sugar ring allows the ribofuranose structure to assume a range of conformations but, once it was realised that the C3′-*endo* configuration is more likely to stabilise a triplex, a range of modifications were explored, with the aim of promoting this conformation.^48,111^ The first generation of BNA is locked nucleic acid (LNA), which consists of a 2′-O, 4′-C methylene bridge that restricts the sugar backbone movement (Fig. 12a) and promotes the formation of an A-form duplex in the binding partner of the LNA strand. This reduced flexibility enhances the stability and selectivity of the TFO strand. It has been reported that including short LNA residues in pyrimidine-motif triplexes will enhance stability due to the significant puckering amplitude.^48^ However, the modification can only be included once in every 2–3 nucleotides; a TFO composed of only LNA modifications does not form triple helices.

A second modification, with an ethylene link (ethylene-bridged nucleic acid, ENA, Fig. 12b) instead of methylene, was proposed to overcome this incorporation limit and allows for the production of fully modified TFOs able to form a triplex.^48,70^ This modification is less restrictive, and therefore allows for a greater variation in the observed LNA sugar pucker, giving more flexibility to accommodate the third strand, which can be composed fully of ENA.^112^

Alternative strategies to modify the sugar component of the TFO without imposing a locked conformation are the addition of an ammonium group to the sugar, 2′-*O*-methylribose (2′-OMe) (Fig. 12c)^113^, or a protonated aminoethyl group at C3′-*endo*, 2′-*O*-aminoethylribose (2′-AE) (Fig. 12d). Both modifications bias the sugar pucker towards C3′-*endo*, favouring the A-form conformation, improving the stability of the TFO towards nucleases and enhancing triplex stability.^114^

Continued development of nucleotide analogues, modified phosphate backbone and sugar-based variants is ongoing. When evaluating a modified TFO, an ideal candidate forms DNA triplexes with a high association rate and remain thermostable, both *in vitro* and *in vivo*. Thus far, modifications have typically been investigated singularly *i.e.*, candidate TFO strands have contained one modification, although this can be at multiple sites within a single strand. Future development should therefore focus on combining modifications to provide a successful outcome in terms of triplex stability and biological function. Indeed, for cellular applications, it must be taken into consideration that the TFO or DNA triplex must initially be delivered into the cell and therefore the hydrophobicity properties must be considered and carefully balanced. The main challenges, however, are to stabilize the triplex at physiological pH, maximise nuclease resistance and finally promote specificity in sequence targeting.

### 4.5 DNA triplexes intercalators and groove binders

A completely different approach to DNA triplex enhancement that does not require chemical modification or solutes is the noncovalent intercalation of a small molecule stabiliser. The latter are molecules, widely studied over the years, which are able to specifically bind DNA triplexes, since they can provide tools to enhance triplex stability and support biological applications. As we have seen, triplex structures are less stable than the duplexes. Specifically, the need for cytosine protonation in the pyrimidine third strand leads to limited triplex stability at physiological pH. For these reasons, intercalation by molecules able to selectively stabilize the triplex structure is of great interest.^96^

**Fig. 13:**
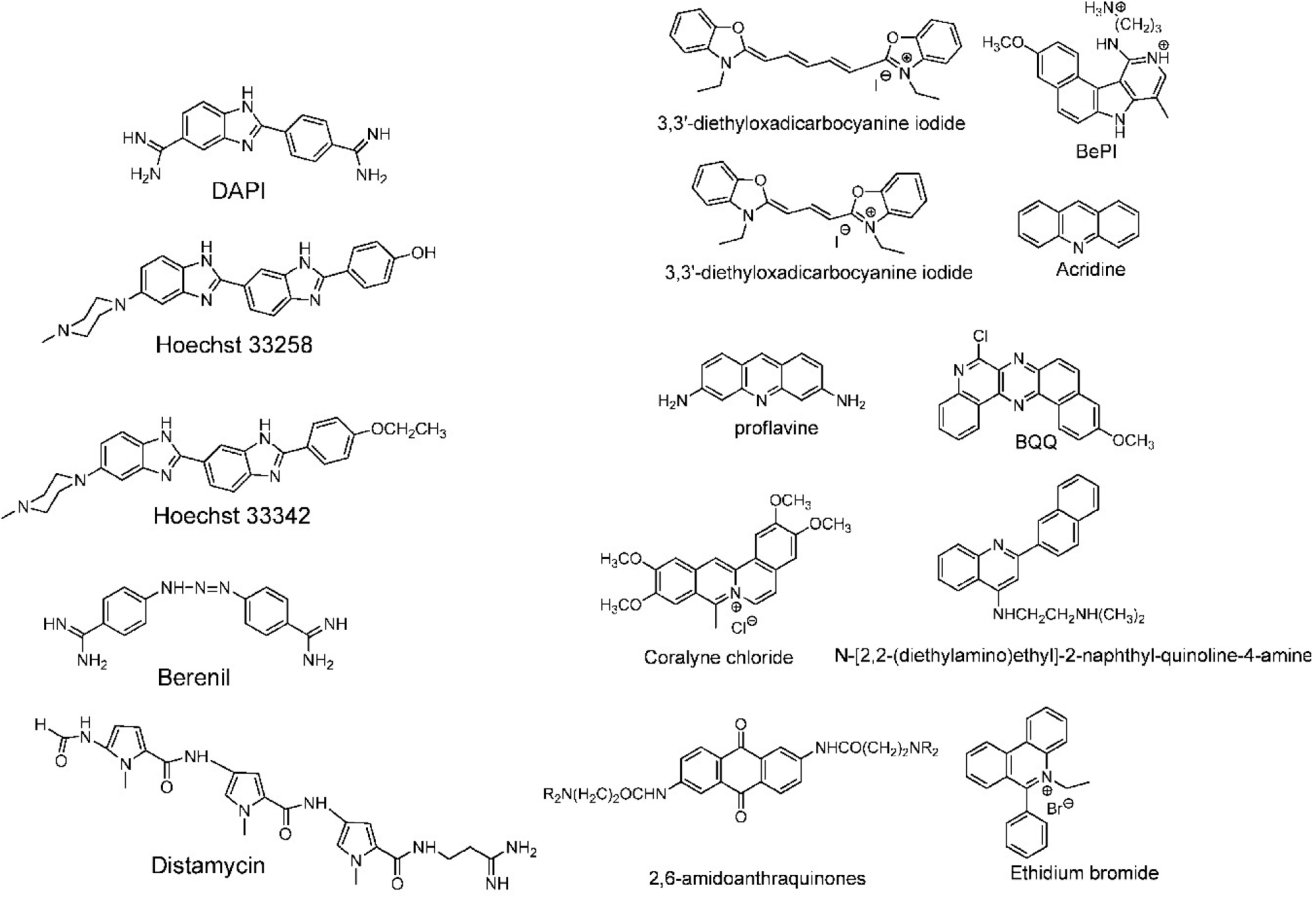
(Left) DNA triplex groove binders and (right) DNA triplex intercalators. Adapted with permission from D. P. Arya, *Acc. Chem. Res.*, 2011, **44**, 134–146. Copyright 2011 American Chemical Society.

For example, the common duplex DNA binder ethidium bromide (EtBr, Fig. 13) can also stabilize a C–G:C structure with a triplex-specific stabilizing effect, due to the electrostatic repulsion between ethidium and cytosine. However, the stabilization of the triplex with ligands will also depend on the concentration of the chosen ligand. It has been reported that two molecules of either EtBr or acridine orange (AO, Fig. 13) in 10-base pair long triplex will stabilise the structure, while a third molecule leads to destabilisation, highlighting that the effect of concentration must be carefully balanced.^115^ Also, the increase of stability, measured as the increase in the triplex melting temperatures, depends on the DNA sequence. The melting temperatures of the 15-mer triplexes were obtained from the hyperchromicity observed at 260 nm upon thermal denaturation. A larger increase in melting temperatures for sequences having A-tract duplex structures was observed by UV spectroscopy, using a ratio of 2 : 1 pyrimidine to purine strand. This large thermal stabilizing effect on dTn·dAn–dTn triplexes is partly due to the intercalators that break up the intrinsic A-tract structure of the underlying duplex.^116^ In fact, the intrinsically rigid and highly propeller-twisted structure of A-tract DNA disfavours triplex formation.^117^ Propidium iodide (PI, Fig. 13) has been reported as a potent stabiliser of the parallel triple helix, with association constant similar to that of PI binding to duplex DNA.^118^ PI was shown to increase the parallel triplex stability after intercalation of three molecules into the triplex, with melting temperature increasing from 21.4 up to 44.4 °C in different media such as Na phosphate buffer, pH 7 and NaCl.^119^

Other DNA triplex binding intercalators include indolocarbazole and benzopyridoquinoxaline derivatives. These provide additional stacking interactions with the pyrimidine strand of the Watson–Crick double helix, resulting in a very efficient and specific stabilizing effect on triple helices and/or in inducing triple helix formation under physiological conditions.^99^

**Fig. 14:**
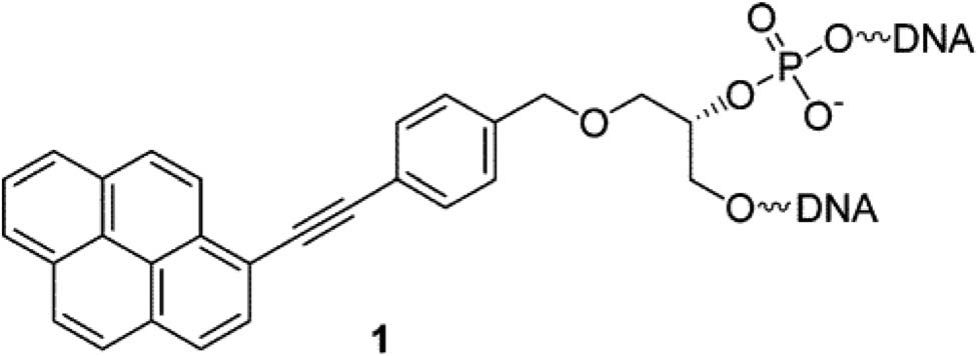
Example of structure of a TINA intercalating unit.^181^ Reprinted with permission from I. Géci, V. V. Filichev and E. B. Pedersen, *Bioconjug. Chem.*, 2006, **17**, 950–957. Copyright 2006 American Chemical Society.

Another class of intercalators able to stabilize the triple helices is the twisted intercalating nucleic acids (TINA) (Fig. 14). These nucleic acids are characterised by the ability to twist around a triple bond. This twisting promotes intercalation within double stranded DNA in order to form triplex DNA. Moreover, it has been demonstrated that these oligonucleotides can discriminate between matched and mismatched sequences of DNA.^120–122^

In this context, it is worth noting that intercalators usually have a stabilising effect on DNA triplexes, whereas minor groove binders will generally destabilise the assembly (Fig. 13). Nevertheless, some aminoglycosides were tested as triplex binders and it was shown that neomycin selectively recognises the triplex Watson–Hoogsteen groove and stabilises it without any effect on dsDNA. This very interesting selectivity may be related to the shape complementarity to the triplex Watson–Hoogsteen groove (the groove formed between the TFO and DNA strand which does not bind to the TFO).^123^ Other minor groove binders that are well exploited are netropsin, spermine and cyclopolyamines.^124^ Psoralen has also been used as it can intercalate efficiently between bases and can provide a covalent linkage by forming an adduct on photoreaction with the stacked pyrimidine.^125–127^

Other reported groove binders are Hoechst 33258, Berenil, DAPI and distamycin A (Fig. 13), however, their stabiliser ability as well as the triplex stability is lower than with neomycin. In this area almost no structural characterisation of triplex–ligand systems has taken place and therefore this is an area which could be the subject of future focus to understand the DNA triplex–ligand molecular interaction.^99,124^

## 5. Applications based on biomolecular approaches

The ability to form a three-stranded complex based on base–base recognition can be exploited to develop biotechnologies suited for diagnosis, prognosis, or disease treatment. Indeed, a modified TFO included in a dsDNA is considered as a potential future for genetic medicine, exploiting sequence-specificity to target genes for manipulation. TFOs have proven to be useful tools, able to alter gene expression and cause genome modification in mammalian cells.^128^ However, several limitations must be overcome to improve their therapeutic value. Often, these applications are restricted because of the low-affinity binding *in vivo* conditions, as well as TFO stability and integrity during cellular uptake. Numerous attempts have been made to modify oligonucleotides and improve these characteristics, as discussed earlier in this article.^96^

The ability of a TFO to inhibit a transcription was demonstrated for the first time with the human c-*myc* protooncogene in HeLa cells. This protooncogene plays a crucial role in normal cell proliferation and programmed cell death. In particular, c-*myc* gene expression is present in cancer cells at an increased level compared to normal cells.^129^ Specifically, after entering the nucleus, TFOs bind to the DNA duplex at the target sequence to form the triple helix, which prevents the polymerase and other transcription factors from initiating transcription. This results in the inhibition of mRNA synthesis from the c-*myc* promoter, demonstrating that the administration of the TFO to the cells can influence the transcription of the c-*myc* gene.^130^

A therapeutic application that was proposed relied on the ability of the TFOs to bind a duplex structure related to the Friedreich's ataxia gene. The formation of the triplex structure stalls the RNA polymerase and decreases the frataxin protein level, which causes the disease. The GAA triplet repeat, which is responsible for the neurodegenerative disease, folds back, forming a triplex structure with the polypurine strand. In this case, disfavouring the formation of the triplex structure could be the key to restore the FXN gene transcription, and therefore regenerate the normal frataxin protein level.^131,132^

Since the TFO should form a DNA triplex along a gene of interest, it is useful to direct a site-specific mutation. Indeed, a psoralen-modified TFO directed to the supF reporter gene, along with UV irradiation in order to allow the cross-linking of the psoralen to the DNA, resulted in a 100-fold increase of mutations, in which 70% are TA to AT transversions. In mammalian cells, chromosomal mutations have been enhanced tenfold after targeting specific genes. Moreover, triplex formation creates a helical distortion to trigger DNA repair by different pathways, *i.e.* involving the nucleotide excision repair (NER) system or homologous recombination (HR).^96^

In addition to induced mutagenesis, another role of the DNA triplex is genome modification based on the recombination strategy. Triplex technology was used to determine whether interstrand cross-links (ICL) could be repaired through homologous recombination (HR). Indeed, a green fluorescent protein reporter forms a triplex with the psoralen-TFO and intercalates through the specific ICL sequence by confirming the HR effect.^133^ Moreover, targeting a specific gene sequence could be used for deleting or replacing sequences on chromosomes. Therefore, a DNA break that happens during the formation of the triplex, stimulates the recombination. To support this notion, a simian virus 40 (SV40) shuttle vector was modified to present psoralen-TFO, then inoculated in human cells, resulting in DNA damage. As consequence, a mutation is induced in a NER/XPA dependent manner.^134,135^ A result obtained with luciferase reporter assays shows that p53 was transactivated when a triplex-forming sequence, introduced *via* plasmid, was formed close to the p53 target sequence.^136^

As reported above in this review, one of the major problems related to TFO application *in vivo* is the instability of the triplex at neutral pH, due to the requirement of cytosine protonation to form the triplex, which is not possible at physiological pH. Different strategies have been studied, such as walled nanotubes (SWNT), to stabilize C–G:C triplexes under physiological conditions. Such studies may facilitate the application of nanomaterials in the artificial control of gene expression and biosensing.^137^ Another interesting and very recent approach proposes to modify the TFOs with the nucleobase 6-amino-5-nitropyridin-2-one (*Z*), which acts as uncharged replacement for the protonated cytosine. By using this method, Rusling obtained stable and selective triplex formation stable at neutral or even slightly basic pH.^79^

**Fig. 15:**
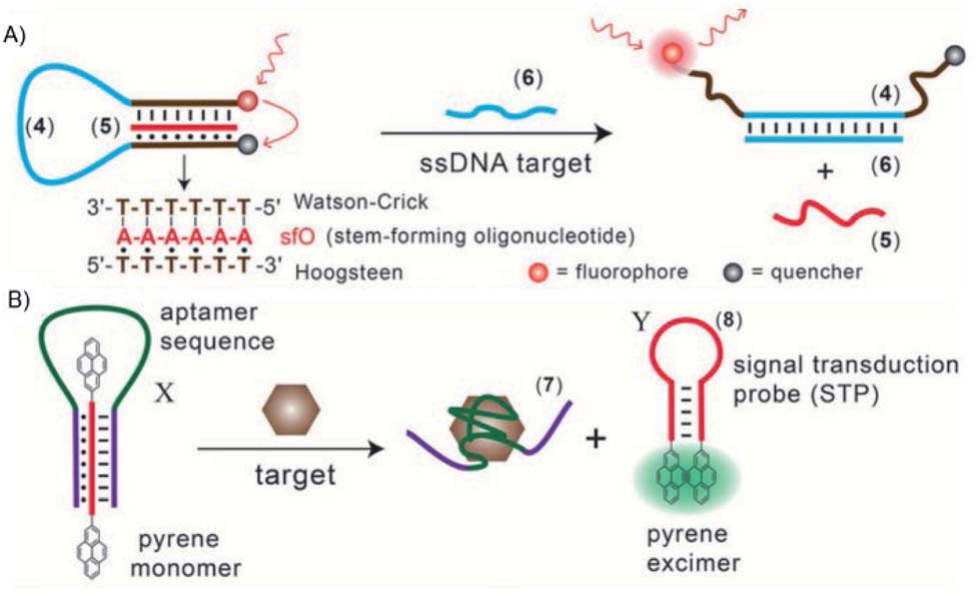
(A) Optical sensor based on hairpin triplex structure (4) of a target gene (6) by the reconfiguration of a fluorophore/quencher-modified triplex DNA hairpin structure and the release of the stem forming oligonucleotide (5). (B) A triplex DNA hairpin moiety (X) containing an aptamer sequence used as an optical aptasensor that binds the target (7) with subsequent formation of a hairpin excited structure (8). Reprinted and adapted from *Triplex DNA Nanostructures: From Basic Properties to Applications* Y. Hu, A. Cecconello, A. Idili, F. Ricci, and I. Willner, pages 15210–15233, Copyright (2017), *Angew. Chem.*

Triplex DNA structures were also used as structure-switching units to trigger a signal, following the recognition of specific targets such as proteins, antibodies, small molecules and pH.^138^ For example, fluorophore/quencher pair molecular beacons are exploited as optical switches to detect pathogens and genetic disorders. These tools can be used with triplex structures. Indeed, a hairpin triplex helix, functionalised with a fluorophore in one edge, and the quencher in the other edge, is reconfigured in an open structure after recognition of the target. The target recognition leads to the opening of the triplex structure and to an increase in fluorescence, due to the spatial separation of the fluorophore and the quencher that were adjacent when the hairpin triplex structure was formed. This idea was applied in the design of a bimolecular triplex helix stem for the analysis of a DNA single strand. The stem containing a T–A·T triplex incorporating a poly-T DNA and a poly-A peptide nucleic acid (PNA) strand was used to increase the stability of a molecular beacon. In this case, after recognition of the target, *i.e.* the single strand of DNA, the formation of the DNA duplex leads to the opening of the triplex structure with an increase in the fluorescence signal (Fig. 15A).^139^

Triplex-based hairpins have also been exploited with a luminescent pair to obtain a sensing platform. This system is characterised by the presence of a pyrene excimer pair attached to the two edges of a linear triplex forming oligonucleotide. Once the hairpin portion of the triplex recognises the analyte, the hairpin is opened and folds around the target molecule. The stem with the pyrenes is thus released, and able to fold into another hairpin structure, causing the contact of the luminescent pair (Fig. 15B). This results in the emission of the pyrene excimer at 485 nm. The emission level is then proportional to the concentration of the target species. This sensing platform has been used for the detection of thrombin, ATP or l-arginamide. All these methods exploit the presence of an anti-thrombin/anti-ATP/anti-l-arginamide aptamer sequence in the triplex-based hairpin. Indeed, many sensors can be designed, but their efficacy depends on reliable opening of the hairpin triplex structure after recognition of the analyte. This could be affected by low sensitivity, so strategies to stabilise the target-recognition sequence are required.^140^

Triplexes can be exploited to detect a specific duplex sequence. The duplex assembly is recognised by a suitable TFO sequence folded into a hairpin loop and containing a fluorophore/quencher pair in proximity to each other. In the presence of the duplex target sequence, the fluorophore and the quencher are separated by the opening of the hairpin structure, leading to an increased fluorescence of the system. This fluorescence increase depends on the concentration of the duplex analyte. This method was applied to detect cancer cells and also non-DNA targets, like the NF-kB p50 transcription factor.^141,142^

Beside the application of triplexes as molecular beacons, triplexes have also been applied as functional units for electrochemical sensors. Electrodes have been functionalised with programmed, redox-labeled DNA structures to obtain a probe attached to the electrode surface. The concept is based on the fact that, when the analyte is present, the binding in between the triplex and the target sequence leads to the formation of a duplex structure. This complex displaces the redox label from the electrode surface, suppressing the electrochemical signal produced by the probe itself. In this way, a quantitative determination of the analyte (*i.e.* DNA, proteins, small molecules, metal ions) is obtained by controlling the voltammetric response.^143^ This method has been applied for the analysis of sequence-specific double strands, adenosine, transcription factors and to detect HIV-1 strains.^144,145^

Similarly, triplexes have been used also as pH probes, exploiting the ability of the oligonucleotides to change the duplex/triplex ratio depending on pH. At around pH 5.0, we have already seen that cytosine bases are protonated, permitting the formation of a parallel triplex structure. This concept has been applied in the development of a construct formed by a long strand with two arms capable of bridging a fluorophore/quencher-functionalised strand *via* the formation of the C–G duplex. In neutral conditions, the fluorophore and the quencher are separated in the medium used. In acidic conditions, the protonation of the cytosines promotes the formation of a triplex structure, causing the proximity of the fluorophore/quencher pair and leading to the decrease of fluorescence intensity.^146^

Another application of the pH dependence of the duplex/triplex structure is the control of aggregation/disaggregation of nanostructures driven by the equilibrium between triplex formation and dissociation. In one example, this equilibrium was used to switch the aggregation/disaggregation of gold nanoparticles (NPs), in a reversible process. The nanoparticles were functionalised with nucleic acids that were partially self-complementary. In neutral conditions, the NPs are separated while in acidic conditions (pH 5.0) the formation of a triplex C^+^·G–C structure leads to NP aggregation. When the system is neutralised, the triplex structures were dissociated and the nanoparticles disaggregated.^147^

In the biomedical field, the trigger release of loads is an important objective that has aroused interest. Stimuli–responsive microcapsules loaded with a substrate and stabilised by DNA shells have been used to specifically release a cargo. Elegantly, the microcapsules are released after enzymatic digestion of the DNA shells. In this context, triplexes have been attached to the microcapsules and used as pH-responsive carriers. For example, QD-loaded CaCO_3_ microparticles, coated with poly(allylamine hydrochloride) (PAH) polyelectrolyte and functionalised with nucleic acid composites containing the caged triplex sequences, were used. The DNA-stabilised CaCO_3_ core was dissolved by adding EDTA. At pH 5.0), the triplex structure is formed, with a subsequent separation of the microcapsules and the release of the QD loads.^148^

Overall, all these findings represent very intriguing and promising steps in the application of TFOs in the biomedical field.

## 6. DNA triplex and related interactions with metal complexes

Transition metal complexes have been investigated in the last decades for a large range of healthcare applications, including diagnosis and treatment of various diseases. Several characteristics are appealing for study with nucleic acids, such as the positive charge, the ability to coordinate directly to Lewis base sites on DNA, the possibility to undergo redox reactions with DNA and to generate reactive oxygen species – an attribute particularly relevant for photodynamic therapy (PDT) – make these systems exceptionally attractive for the development of new therapeutics.^149^

Since the serendipitous discovery of cisplatin and its ability to covalently bind duplex DNA,^150^ many metal complexes have been studied to obtain compounds with less side effects than cisplatin and an improved and more selective toxicity towards cancer cells. In parallel, other approaches to the use of metal complexes for targeting DNA in different ways have been developed.^151–153^ Very interestingly, metal compounds can also be exploited with non-canonical DNA structures, to stabilise these structures and/or to functionalise them for a specific application, as presented in this section.

Early attempts were made to introduce Ag(i)-based complexes as artificial nucleosides to stabilise DNA triplexes through metal complexation. The incorporated Ag(i) complex significantly stabilised the DNA duplex and triplex by introducing a pair of pyridine nucleobases in the middle of the sequence. The nitrogen of the pyridyl complex coordinates with Ag(i) at the centre of the triplex, stabilising the triplex structure.^154^ Although it is not an independent molecule that intercalates in the DNA triplex, it is noteworthy that OsO_4_^−^ bipyridine stabilises the triplexes by protecting the thymine from being disrupted. In the study, it was observed that intercalation caused a thymine base to flip out of the DNA helix. When the complex was added, the thymine was protected from this disruption.^155,156^

The ability to specifically recognise a non-conventional DNA structure is a very powerful tool to increase specificity in targeting biomolecular sites. For example, tetracationic supramolecular helicates such as [Fe_2_L_3_]^4+^, formed from Fe^2+^ ions wrapped by three bis-pyridylimine organic strands, were used in a new approach for synthetic DNA recognition. Intriguingly, one of the compounds (L = C_25_H_20_N_4_) recognised a three-way junction in duplex DNA, giving a unique hydrophobic binding site characterised by a triangular shape. The structure was determined by X-ray crystallography. This result gave information on the existence of DNA binding modes of metal-based drugs that differ from the most common ones (*i.e.*, covalent bond, intercalation, major groove binding, minor groove binding and sugar–phosphate backbone binding).^157^

Bulges are sites of DNA where one or more nucleotides are not paired within the double helix. These unpaired nucleotides arise after replication and recombination errors or after carcinogen-induced DNA damage. They are believed to play an important role in various diseases such as cancer, Alzheimer and muscular dystrophy. Thus, DNA sequences containing a bulge are an important target for developing potential therapeutic drugs. Also, small molecules able to target DNA bulges are particularly interesting for their use as potential therapies. The interaction of the above-cited compound [Fe_2_(C_25_H_20_N_4_)_3_]^4+^ with bulged DNA was studied by DNA melting temperature and gel electrophoresis assays to evaluate the binding affinity of this helicate for various DNA bulges. Both enantiomers of the compound bind to bulges containing two or more unpaired nucleotides. Moreover, this compound had higher binding affinity for bulges containing unpaired pyrimidines and/or flanking pyrimidines. It is suggested that the bulge allows the triangular prismatic motif necessary to accommodate the helicate. This is an example of another uncommon DNA structure that is specifically recognised by [Fe_2_L_3_]^4+^ supramolecular helicates.^158^ Brabec and co-workers described a class of dinuclear Fe^II^ triplex-forming metallohelices able to specifically recognize and stabilise DNA bulges of different size and composition. The compounds preferably bind the DNA bulges instead of double-strand DNA. Their binding affinity showed to be dependent on the individual metallohelices, the bulge size and the bases present in the bulge loop. In particular, pyrimidine-containing bulges are preferred compared to the purine-containing ones. These compounds were shown to have the ability to stabilise the bulge containing sequences. In fact, an increased thermal stability was obtained with DNA bulges containing three or more unpaired adenines or two unpaired thymines, indicating a stabilising effect.^159^

**Fig. 16:**
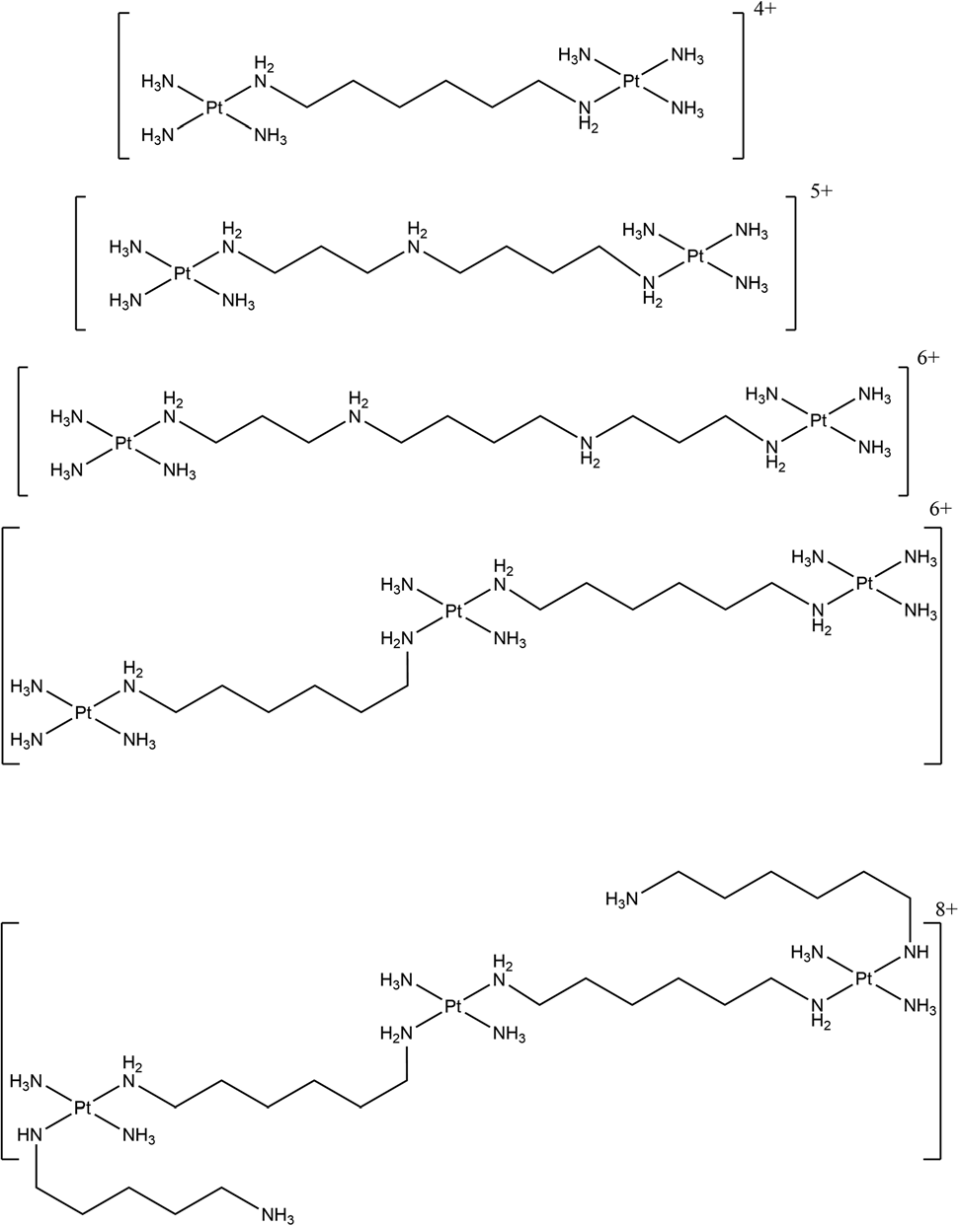
Chemical structures of the various SI-PPCs.

A range of antitumour substitution-inert polynuclear platinum complexes (SI-PPCs) have been studied as small molecules able to recognise, bind and stabilise the triplex structure of DNA and RNA (Fig. 16).

This class of compounds bind DNA through noncovalent interactions, in particular by “phosphate clamp”, a mode of DNA–ligand recognition different from the intercalative or minor groove binding. They had the ability to inhibit DNA synthesis by DNA polymerase when the DNA sequences used are prone to form pyrimidine- and purine-motif triplex DNAs. It was suggested that these compounds act as very effective stabilisers of triplex DNA and that they can play a stabilising role in triple-helical DNA. The results from a *Taq* DNA polymerase assay showed that the pyrimidine-rich template used for the experiment does not permit the primer extension when the SI-PPCs compounds are present. This indicate that the compounds stabilise or form a DNA topology that impedes DNA polymerisation. Interestingly, the formation of the DNA triple helix is not stopped in the absence of the compounds and a displacement of TO (which intercalates with high-affinity in triplex structures) takes place when the SI-PPCs are present. This indicates the ability of the Pt-derivatives to form a complex with triple-helical DNA. It was suggested that the ability to stabilise the triplex structure plays a crucial role in the cytotoxicity of this class of compounds. This is particularly important since nucleotide sequences able to form a triplex structure are present in natural DNA, preferentially near regulatory regions.^160^

Moreover, the ability of these class of compounds to inhibit the reverse transcription in RNA template prone to form a triplex structure was described. In particular, the ability of a class of SI-PPCs to inhibit DNA synthesis by reverse transcriptase was evaluated. A purine-rich primer and a pyrimidine-rich RNA template able (TFT) or non-able (SST) to form triplex structures were annealed together and the reverse transcriptase activity was checked by several biophysical techniques. UV melting studies were used to prove that the TFT annealed with the primer formed a triplex structure, showing a biphasic transition in the melting curve, characteristic of a triplex structure. Moreover, the primer extension was allowed in a reverse transcriptase assay without of SI-PPCs, proving that the triple helix formation does not hamper the reverse transcriptase to extend the primer. On the contrary, in the presence of SI-PPCs, the reverse transcriptase ability to extend the primer annealed with the RNA templates was reduced. This inhibition, related to the presence of the platinum compounds, depends on the charge of the compounds and on their size. Moreover, the inhibiting activity in TFT was higher than in SST, suggesting that SI-PPCs can preferentially recognise, stabilise and inhibit the reverse transcription in RNA template prone to triplex formation rather than in SST. Overall, the ability to bind nucleic acids and inhibit protein–RNA triplex interaction is a very promising extension of the biological activity of this class of compounds.^161^

**Fig. 17:**
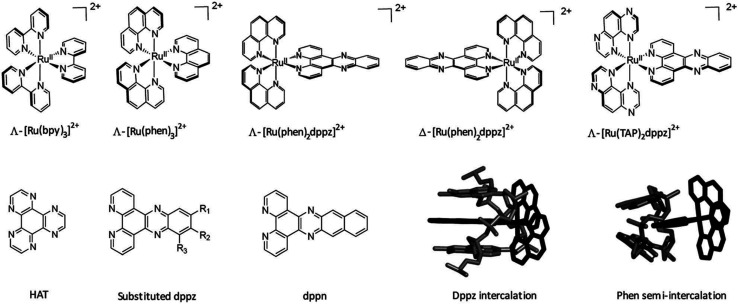
Important ruthenium complexes and binding modes^174^ Reproduced from Cardin C. J., Kelly J. M. & Quinn S. J. Photochemically active DNA-intercalating ruthenium and related complexes-insights by combining crystallography and transient spectroscopy. *Chem. Sci.***8**, 4705–4723 (2017).

A wide range of octahedral ruthenium(ii) complexes have been investigated for potential biomedical uses, making use of the slow rate of ligand exchange for this electron configuration, multiple and accessible oxidation states, positive charge, and ability to mimic iron in the physiological environment.^162^ Ruthenium complexes have been associated with reduced side effects in clinical trials when compared to drugs containing other metals, such as platinum.^163^ Ru(ii) polypyridyl complexes are notable for their favourable photophysical and photochemical properties, such as visible light absorption (lower energy than 400 nm) due to metal-to-ligand charge transfer (MLCT),^164^ and particularly important for the application of such compounds in PDT. This medical technique is based on the use of an ideally non-toxic molecule, called photosensitizer (PS), which is activated by light to produce singlet oxygen with a lifetime in metabolically healthy cells of ∼3 μs just at the site of irradiation, obtaining therefore a high spatial and temporal selective treatment.^165,166^ Indeed, by varying the ligand set, Ru-based complexes can be tailored not only to obtain desired photophysical and photochemical properties in the PDT application window, but also to improve their DNA binding.^167^ Different Ru-polypyridyl compounds have been studied for their ability to intercalate in the DNA by π–π interaction between the aromatic ligands and DNA π-stack (Fig. 17). In the next section, we will present some examples, from the very large range already known, of Ru complexes with interesting photophysical and photochemical properties for the application in DNA binding studies, with special attention to actual or potential triplex DNA binding.^168^

**Fig. 18:**
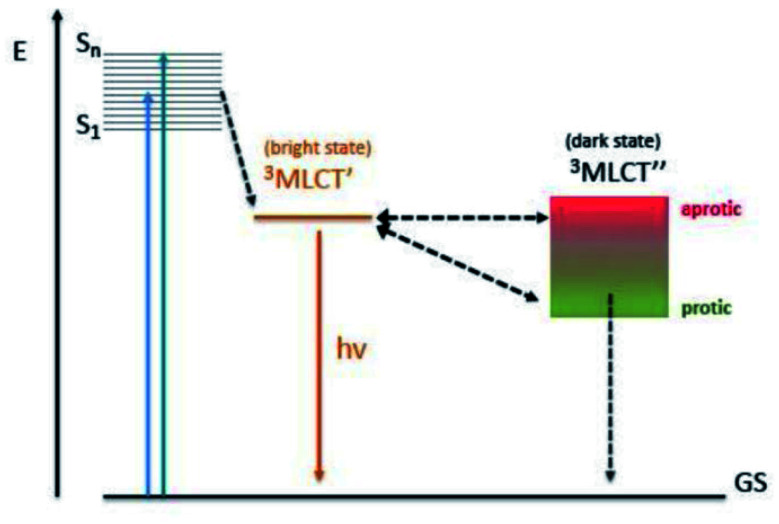
Jablonski diagram indicating the electronic transition from the excited to the ground state, depending on the solvent.^182^ Reproduced from Di Pietro M. L., La Ganga G., La Nastasi F. & Puntoriero F. Ru(ii)-dppz derivatives and their interactions with DNA: thirty years and counting. *Appl. Sci.***11**, (2021).

A series of Ru(ii) complexes with the 1,12-diazaperylene (DAP) ligand of the type [Ru(bpy)_2_(DAP)]^2+^, [Ru(bpy)(DAP)_2_]^2+^, [Ru(bpy)(DAP)_3_]^2+^ (bpy = 2,2′-bipyridine) was shown to intercalate into calf thymus DNA. The DAP ligand is characterized by an extended π-system and a large surface area to improve the DNA intercalation. Because of the lack of water solubility of the bis- and tris-DAP species, thermal denaturation experiments were performed only with the more water-soluble compound [Ru(bpy)_2_(DAP)]^2+^, showing that this compound can stabilise calf thymus DNA with an efficiency comparable to that of ethidium bromide. Moreover, photocleavage of pUC18 supercoiled plasmid was observed in the presence of [Ru(bpy)_2_DAP]^2+^ after irradiation with *λ* > 395 nm for 30 min. The absence of photocleavage in a deoxygenated water environment demonstrated that the ^1^O_2_ species is involved in the photoreactivity with DNA.^167^ As long ago as 1990 the “light-switch” effect of the compound [Ru(bpy)_2_(dppz)]^2+^ (dppz = dipyrido[3,2-*a*:2′,3′-*c*]phenazine) was demonstrated by Barton and co-workers, describing this compound as a highly sensitive spectroscopic reporter of double helical DNA. They demonstrated that this compound displays luminescence only when intercalated into the duplex structure *via* the planar aromatic ligand dppz. It was shown that after intercalation between DNA base pairs the compound displays an intense luminescence activity, quenched in aqueous solution.^169,170^ An accepted explanation is that [Ru(bpy)_2_(dppz)]^2+^ has a non-emissive (dark) MLCT low-lying excited state involving the phenazine moiety of the dppz ligand, and another emissive (bright) MLCT state related to the bpy part of the dppz ligand. In aqueous solution the dark state is favoured being at lower energy compared to the bright state. On the contrary, when intercalated into DNA, the dark state gets closer in energy to the bright state, allowing thermal population and increasing the emission (Fig. 18). The DNA duplex in which the [Ru(bpy)_2_dppz]^2+^ is intercalated prevents the quenching effect of the aqueous solution, resulting in a luminescence effect. Further investigation has demonstrated that after binding the DNA *via* intercalation, also the [Ru(bpy)_2_dppz]^2+^ compound can trigger the photocleavage of pUC18 plasmid DNA in presence of O_2_ (*λ*_irr_ > 455 nm, 15 min).^171^ In 1992, the light-switch effect of both [Ru(bpy)_2_dppz]^2+^ and [Ru(phen)_2_(dppz)]^2+^ was reported as function of the nucleic acid sequence and conformation. Indeed, the strongest luminescence effect was observed when the greatest amount of overlap between the nucleic acid structure and the complex was involved, such as when one of these complexes intercalates into triple helices. In fact, an increased luminescence was observed when the two compounds were bound to the triple helical assembly, permitting the dppz ligand to be better shielded from water by the extended surface area of the triplex. Subsequently, a detailed analysis by Choi *et al.* using separated Λ and Δ enantiomers showed that both compounds can bind to a poly(dT × dA–dT) triplex, displaying an increased luminescence compared to the duplex, assumed to be due to the larger surface area of the triplex that better protects the intercalating ligand dppz from water. This better protection and higher luminescence give a useful diagnostic of triplex formation. At the time of these solution studies, there was no clear structural evidence for any binding mode of these complexes to nucleic acids. Despite the third strand, access for intercalation is possible *via* the major groove, as has been proposed.^172^ Detailed studies with separate enantiomers have elucidated by linear and circular dichroism that the Ru complexes with dppz and dppn (dppn = benzodipyrido[3,2-*a*:2′,3′-*c*]phenazine) as ligands are able to intercalate between the nucleobases of a T–A:T triplex in the minor groove. These authors made a detailed study of the bound chromophore orientation, and concluded that, especially for the Λ complexes, the triplex binding mode had a close resemblance to that seen with duplexes. Very interestingly, the stabilisation of the third strand is related to the nature of the third phenanthroline, showing a stabilizing effect that increases in the order phen < dppn < dppz (phen = 1,10-phenanthroline). Intriguingly, the stabilising effect is not related to the size of the ligand.^173^

At the time of that publication, no structural data on duplex binding by these compounds was available. The later demonstration that the dppz chromophore intercalated exclusively from the minor groove implies that this would also be true with triplexes.^174^ Thereafter, numerous studies have confirmed these interesting features, demonstrating the possible value of this class of compounds as photoluminescent probe for bioanalysis and application in PDT.^164^

Ru(ii) complexes linked to triplex forming oligonucleotides could be used as photosensitisers in site-specific damaged DNA, as demonstrated by Héléne and co-workers. In fact, the complex [Ru(phen)_2_dppz]^2+^ attached to the oligonucleotide and intercalated in the DNA formed a stable triplex. Different behaviours were observed between the two enantiomers of the compound, in fact the luminescence of the Δ enantiomer linked to HIV-T oligonucleotide increased by 6–10 times, while no enhancement was observed with the Λ enantiomer. The Δ enantiomer of the compound [Ru(phen)_2_dppz]^2+^ linked to the 5′-phosphate group of the oligonucleotide by phenanthroline binds the DNA duplex in a sequence-specific way. The proposed mechanism is the formation of the triplex and the intercalation of the dppz ligand into the DNA molecule, leading to the stabilisation of the structure and to an enhancement of the fluorescence. Once again, the photophysical properties of ruthenium compounds such as the ability to photocleave, long-distance electron transfer and luminescence can be exploited for application in antigene-therapy or as photosensitiser for photodamage of the DNA by triple helix formation.^175^ Indeed, the [Ru(phen)_2_dppz]^2+^ complex is reported to successfully bind the DNA double helix, so that this property can be exploited to stabilise the triplex by conjugation of the complex to the 5′-end of a TFO. Importantly, the triplex formed by a TFO functionalised with [Ru(phen)_2_dppz]^2+^ showed an increased stability by thermal denaturation compared to the triplex formed by the same unmodified oligonucleotide, with a Δ*T*_m_ = 12 °C. This indicate that the unmodified oligonucleotide forms less stable triplexes than the nucleotide decorated with the ruthenium complex.^168^ The strong aromatic character of the dppz ligand allows for the intercalation both in duplex and triplex DNA, lying parallel to the triplex bases and intercalating into the minor groove of the triplex. Notably, the whole triplex structure is stabilised by the intercalation of the Ru-dppz complex bound to the TFO.^176^

Therefore, Ru polypyridyl derivatives are of great interest to obtain a stabilising effect on triplexes and to selectively cleave DNA by exploiting the high binding specificity of TFO and the photophysical properties of the ruthenium derivatives linked to the TFO.

A library of Ru(ii) complexes with halogenated dppz ligands was screened against several biological molecules, such as proteins, ssDNA, dsDNA, DNA triplexes and DNA G-quadruplexes to understand the main factors influencing luminescent behaviour. It was proposed that (i) intercalation in the DNA structure of these compounds mainly depends on the changes of the halogenated substituent on the dppz ligand, (ii) the luminescence is increased in the presence of DNA structures but not in the presence of hydrophobic non-DNA structures such as BSA (iii) the π stacking surface area influences the luminescence. Indeed, after studying a panel of different substituents on the dppz ligand, more luminescence effect was detected with the compound [Ru(bpy)_2_dppz-11,12-Br]^2+^ in the A–T:A triplex and in intrastrand G-quadruplexes compared to intercalation into the DNA duplex. The authors suggest that large Br atoms in positions 11 and 12 prevent the complex from fully intercalating in the DNA duplex, causing the phenazine N atoms to be partially exposed to water, resulting in increased luminescence quenching. The luminescence was enhanced by 89× in the presence of DNA triplexes compared to that in buffer alone. Moreover, this compound has also shown a 2.8× higher luminescence when bound to G-quadruplexes compared to DNA triplexes, confirming that the π stacking surface area plays an important role in increasing the luminescence.^177^ More structural studies are required to understand if this effect is due to the structure itself or to the DNA sequences.

DNA triplexes have also been used as part of an Enhanced Chemiluminescence (ECL) biosensor approach to detect the presence of adenosine in serum (Fig. 18). The ECL based on [Ru(bpy)_3_]^2+^ complexes are used to detect a large number of analytes with different percentages of selectivity and sensitivity. Those characteristics change based on different elements that are part of the ruthenium complexes. Nevertheless, the advantage is to work with an approach that completely avoids radioactive labels with the limit of detection that is low and simple to use. To quantify the presence of adenosine in serum, the method is based on an aptamer, attached on the surface of a gold electrode with an ECL signal marker composed of [Ru(bpy)_3_]^2+^ forming the first DNA strand. The other strand used as a quenching probe binds a ferrocene carboxylic acid (FcA) at the 5′ end. A complex is formed with a third strand, complementary to the quencher, and coralyne chloride as binder. This complex is stable until the concentration of the adenosine increases. At this point the first strand assumes a hairpin configuration generating an intense luminescence due to the ruthenium complex and the absence of the FcA activity. This technique based on a DNA triplex has a more sensitive adenosine detection compared to the DNA duplex-based sensor.^178^

## 7. Conclusions

DNA triplexes are non-canonical structures that together with other unusual configurations, such as *i*-motif or quadruplexes, are part of the molecular biology field that is yet to be exploited. Triplexes possess large diversity in terms of stability, distortion, and environmental conditions required for the formation. In order to exploit the DNA triplex for biological uses, numerous ligands have been designed over the years to functionalise these structures and enhance their stability in physiological conditions. Whilst multiple metal-based compounds have been developed to interact with DNA triplexes, Ru(ii) polypyridyl compounds are of significant interest due to their photophysical, electronic and biological properties.^164^ Many interesting and promising results have been obtained. However, investigations that cover the role of ruthenium complexes in DNA triplexes are very limited. More studies are required to overcome the difficulties related to their chemical and cellular properties and increase the possibility of medical applications.

## Author contributions

MDP and AA contributed equally to this work. The literature review was undertaken by MDP and AA with input from CJC, GG and JPH. MDP and AA led the writing of the manuscript with input from CJC, GG and JPH.

## Conflicts of interest

There are no conflicts to declare.

## Supplementary Material
